# Protein Assembly Modulation: A New Approach to Amyotrophic Lateral Sclerosis (ALS) Therapeutics

**DOI:** 10.33696/Neurol.5.103

**Published:** 2024

**Authors:** Shao Feng Yu, Kumar Paulvannan, Dennis Solas, Anuradha F. Lingappa, Ana Raquel Moreira, Shriya Sahu, Maya Michon, Amanda Macieik, Danielle Goldsmith, Nicholas DeYarman, Suguna Mallesh, M. Dharma Prasad, Claudia Maios, Kai Ruan, Giulio S. Tomassy, Elizabeth Jensen, Emma McGuirk, Verian Bader, Andreas Mueller-Schiffmann, Jonathan C. Reed, Jaisri R. Lingappa, Vinod Asundi, Shi Hong, Steve Jacobsen, Nicholas Brandon, Lyle Ostrow, Tom Lloyd, J. Alex Parker, Kim A. Staats, Justin Ichida, James C. Dodge, Debendranath Dey, Carsten Korth, Suganya Selvarajah, Vishwanath R. Lingappa, Jeffrey Rosenfeld

**Affiliations:** 1 Prosetta Biosciences, Inc. 670 5th St San Francisco, CA 94107, USA; 2 University of Montreal, Quebec, Canada; 3 Current address Department of Neurology, Division of Biological Sciences, The University of Chicago, Chicago, IL, 60637, USA; 4 Biogen, Cambridge, MA 02142, USA; 5 Sanofi, Genomic Medicine Unit, Waltham, MA 02451, USA; 6 Sanofi, Framingham, MA 01701, USA; 7 Department of Neuropathology, University of Dusseldorf, Germany; 8 University of Washington, Seattle 98109, USA; 9 Discovery, Neuroscience, BioPharmaceuticals R&D, AstraZeneca, Boston, MA, USA; 10 Temple University, Lewis Katz School of Medicine, Philadelphia, PA 19140, USA; 11 Department of Neurology, School of Medicine, Johns Hopkins University, Baltimore, MD 21205, USA; 12 University of Southern California, Los Angeles, CA 90033, USA; 13 Department of Physiology, University of California, San Francisco, CA 94116, USA; 14 Department of Neurology, Loma Linda Medical Center, Loma Linda CA 92354, USA

**Keywords:** Affinity chromatography, Allostery, Molecular neuroscience, Neuro-degeneration, Phenotypic screen, Photocrosslinking, Protein assembly, Small molecule drug discovery, Structure activity relationship, Translational neuroscience

## Abstract

Amyotrophic Lateral Sclerosis (ALS) is a devastating and progressive neurodegenerative disease with a complex, multifactorial pathophysiology, culminating in death of motor neurons. We introduce a new mechanism of ALS pathogenesis via study of a novel drug-like small molecule series that targets a subset of protein disulfide isomerase (PDI) within a previously largely unappreciated transient and energy-dependent multi-protein complex enriched for proteins of the ALS interactome. This drug, found by a novel phenotypic screen, has activity in cellular models for both familial and sporadic ALS, as well as in transgenic worms, flies, and mice bearing a diversity of human genes with ALS-associated mutations. The hit compound was initially identified as a modulator of human immunodeficiency virus (HIV) capsid assembly in cell-free protein synthesis and assembly (CFPSA) systems, with demonstrated antiviral activity against infectious HIV in cell culture. Its advancement for ALS-therapeutics, subsequent separation of activity against HIV and ALS into separate chemical subseries through structure-activity-relationship (SAR) optimization, and identification of the drug target by affinity chromatography as shown here, may provide insights into the molecular mechanisms governing pathophysiology of disordered homeostasis relevant to ALS.

## Background

### Amyotrophic lateral sclerosis

Aberrant protein aggregation is a common pathophysiologic mechanism implicated in a variety of neurodegenerative disorders [1,2]. In the case of ALS, a serious neurodegenerative condition primarily involving motor neurons, it is generally accepted that a cellular manifestation of disease is the mislocalization and/or aggregation of transactive DNA-binding protein of 43 kDa (TDP-43) [3–8]. In healthy individuals, TDP-43 is localized to the cell nucleus [9,10]. However, at autopsy, almost all cases of ALS studied have shown TDP-43 mislocalized to the cytoplasm to varying degrees [5,6,9,10]. The mis-localized TDP-43 is found in aggregates, typically co-localized with stress-granule proteins [7,11,12]. For poorly understood reasons, the end result is selective death of motor neurons.

ALS is a challenging disease to study, diagnose, and treat in part because it is heterogenous in phenotype and progression, both in cells and clinically, in individuals living with ALS [13–15]. The overwhelming majority of ALS cases are sporadic, meaning that the ALS patient does not have a family history of the disease [16]. Among sporadic ALS cases, age of onset, manifestation, and disease progression are variable. While in some cases patient’s symptoms worsen quickly, in another subset they progress slowly [13,15]. A small subset of ALS is familial, for which specific gene mutations have been identified [16–18]. The proteins encoded by these genes, and those with which they interact, comprise the ALS interactome [5,19]. The specifics of how exactly these gene products work together normally, or malfunction to cause ALS, have not been established [20]. Likewise, specific toxins have been implicated in the increased incidence of ALS-like syndromes, but their significance for sporadic ALS remains unknown [21,22].

The identification of specific genes in familial ALS has made possible the construction of transgenic animal models that show key phenotypic manifestations of ALS. *Caenorhabditis elegans* is a simple worm model with a total of 302 neurons [23]. The *C. elegans* models for ALS have human TDP-43 or FUS gene mutations or C9orf72 repeat expansions, which show neuronal degeneration [24]. Wildtype *C. elegans* are able to swim in liquid media, but when transgenic *C. elegans* bearing human ALS-causing gene mutations are placed in liquid medium they display swimming-induced paralysis likely in response to stress [24]. *Drosophila melanogaster* is a more complex animal model with nearly three orders of magnitude more neurons [25]. *D. melanogaster* with human C9orf72 repeat expansion transgenes show retinal neurodegeneration and developmental lethality [26]. Mouse models for ALS, where TDP-43 mutations, C9orf72 repeat expansions, or SOD1 mutations are introduced as transgenes, all develop paralysis and neurodegeneration [27]. We will use a number of these diverse cellular and animal models for our studies.

### Protein assembly

Proteins are made individually on ribosomes, but often are found in multi-protein complexes [28] and thus newly synthesized proteins must rapidly associate with their correct partners despite the crowded nature of the cytoplasm [29].

Viral capsid formation is perhaps the most robust protein assembly pathway known [30]. Long viewed as occurring through spontaneous self-assembly [31], a body of literature suggests kinetic control over the thermodynamic endpoint of capsid formation through host-mediated catalysis [32–34]. We hypothesized that protein aggregation diseases, including ALS, might be related to disordered protein assembly [35]. If viral capsid formation is host-catalyzed, as suggested by the formentioned citations, then perhaps that is the case more generally for protein assembly, including for host multi-protein complexes.

A second dimension of this puzzle is that many regulatory proteins are found to be involved in literally thousands of different protein-protein interactions [36]. Thus, not only must multi-protein complex interactions happen rapidly during or immediately after protein synthesis, but the correct interactions must occur. How these interactions are achieved selectively and faithfully has been a long standing mystery that has likely confounded drug discovery efforts, and for which diverse mechanisms have been proposed [37–40].

It is only a small further extension of the hypothesis to suggest that protein aggregation diseases reflect dysregulation of normal catalyzed protein assembly events. In the case of viral infection, the process of repurposing host machinery in a specific way for capsid formation has been fine-tuned by natural selection through deep evolutionary time. In the case of our hypothesis on non-viral protein aggregation diseases, that process is not likely programmed in onset, but once initiated could be just as inexorable, albeit perhaps on a longer time frame (decades for neurodegeneration rather than hours for viral infection). A corollary is that drugs restoring homeostasis by targeting host machinery that had been repurposed by viruses to drive catalyzed capsid assembly may also be therapeutic for specific diseases of protein aggregation, such as ALS, if those diseases represent dysfunction of the same or related protein assembly machinery. Small molecules active against host machinery catalytic for viral capsid formation then, may be a good starting point for advancement of protein assembly modulators for non-viral diseases.

Cell-free protein synthesis is a powerful tool because it recreates key events of gene expression, starting with mRNA translation, in a manner with substantial fidelity to events occurring in living cells – but in slow motion and amenable to experimental manipulation (e.g. subcellular fractionation and functional reconstitution) [41]. For this reason, cell-free protein synthesis made possible the deciphering of the genetic code [42,43] and our current understanding of protein targeting to intracellular compartments [44]. We utilized a variation on the theme of this approach to construct cell-free protein synthesis and assembly (CFPSA) systems that allowed us to define a host-catalyzed pathway for viral capsid formation [45,46]. That system was then adapted into a moderate throughput phenotypic drug screen to identify small molecules that block formation of viral capsids [32,33,47,48]. A diverse, drug-like 150,000 compound library was screened for small molecules with protein assembly modulating properties with respect to capsid formation, for each of the viral families causing significant human disease. From this screen a small numbers of structurally unrelated hit compounds were identified for each viral family [30]. These “protein assembly modulator” compounds were validated against infectious viruses [32,33,47–49]. Subsequently, they were tested in cellular and animal models for a diversity of nonviral disease, with success [47,49] analogous to the studies reported here for ALS.

Hit compounds were advanced through medicinal chemistry analog synthesis prior to use of advanced compounds as affinity ligands for identification of drug targets [50]. The data from drug resin affinity chromatography experiments showed that, for multiple structurally-unrelated classes of assembly modulators, the compounds identified from our screen appeared to target multi-protein complexes comprised of proteins implicated in specific disease pathogenesis.

Proteins identified in the target complexes included known allosteric regulators such as 14-3-3, Protein Disulfide Isomerase (PDI), and KAP1/TRIM28 [47]. The literature on allosteric regulator proteins such as 14-3-3 and KAP1 shows that their impact on health is context-dependent. In some circumstances, they are associated with poor prognosis and extreme pathology. In other circumstances, the same proteins are implicated in maintenance of homeostasis. Likewise, an assembly modulator whose activity was validated for prevention of tau hyperphosphorylation in the Alzheimer’s transgenic mouse, targets an allosteric site rather than an active site, on the protein MIF [49].

Our working hypothesis is that allosteric sites govern the multi-protein complexes targeted by protein assembly modulators in ways that allow the compounds to reverse disease-associated changes. We’ve seen that treatment with assembly modulators changes the composition of their target multi-protein complexes. In some cases, treatment with protein assembly modulators appears to not just block the pathological process, but to actually restore key features of homeostasis. This includes restoration to the multi-protein complex of key regulators of autophagy such as p62/SQSTM1, that had been lost in disease [47,48].

### The relationship of viruses to neurodegenerative diseases

Applications of these compounds, originally identified as modulators of viral assembly, in the realm of neuroscience in general and ALS in particular, were prompted by an additional consideration. There is a long and enigmatic history of association of particular viruses with specific neurodegenerative diseases. Thus, influenza has long been associated with Parkinson’s Disease [51,52], herpesvirus infections with Alzheimer’s Disease [53], and endogenous retroviral activation is observed in ALS [54]. Moreover TDP-43 whose aggregation is implicated in pathophysiology of ALS, also functions as a regulator of HIV-1 transcription [55]. Finally, the emergence of cognition through natural selection may have been due, in substantial measure, to retroviral-mediated genetic novelty [56].

The forementioned hypotheses provided a biologically plausible rationale for the line of investigation pursued here [35,49]. Specifically, we used protein assembly modulator compounds active against capsid assembly of HIV, a retrovirus, as the starting point for our ALS counterscreen [33,57]. The results provide a new framework for understanding the underlying pathophysiology of ALS. We show that a chemical series originally identified and validated against HIV, apparently also corrects a molecular-level defect responsible for TDP-43 mislocalization, with therapeutic efficacy in multiple models of ALS.

Importantly, this chemical series has been progressed to a subseries lacking anti-HIV activity and with further enhanced therapeutic potency for treatment of ALS. Thus, the anti-viral target and the anti-ALS target, while related, appear distinct. We suggest these results reflect a restoration of homeostasis, maintenance of which may be the normal function of the transient, energy-dependent multi-protein complexes targeted through allosteric sites by protein assembly modulator drugs, across therapeutic areas. Our studies presented here provide an initial assessment of the relevance of assembly modulator compounds for therapeutics of ALS.

## Results

### Activity of HIV-assembly modulating compounds in cellular models of ALS

A phenotypic screen was established for identifying drug-like small molecule compounds which inhibited HIV capsid assembly in a CFPSA system [33]. The biological relevance of the CFPSA-derived protein assembly modulators was demonstrated by the activity against infectious HIV in cell culture [33,47] including of analogs of a Tetrahydroisoquinoline (THIQ) chemical series (see Supplemental Figure 1A for structure and synthetic scheme of PAV-073, an advanced ALS-selective molecule within this series) [33].

**Figure 1.**
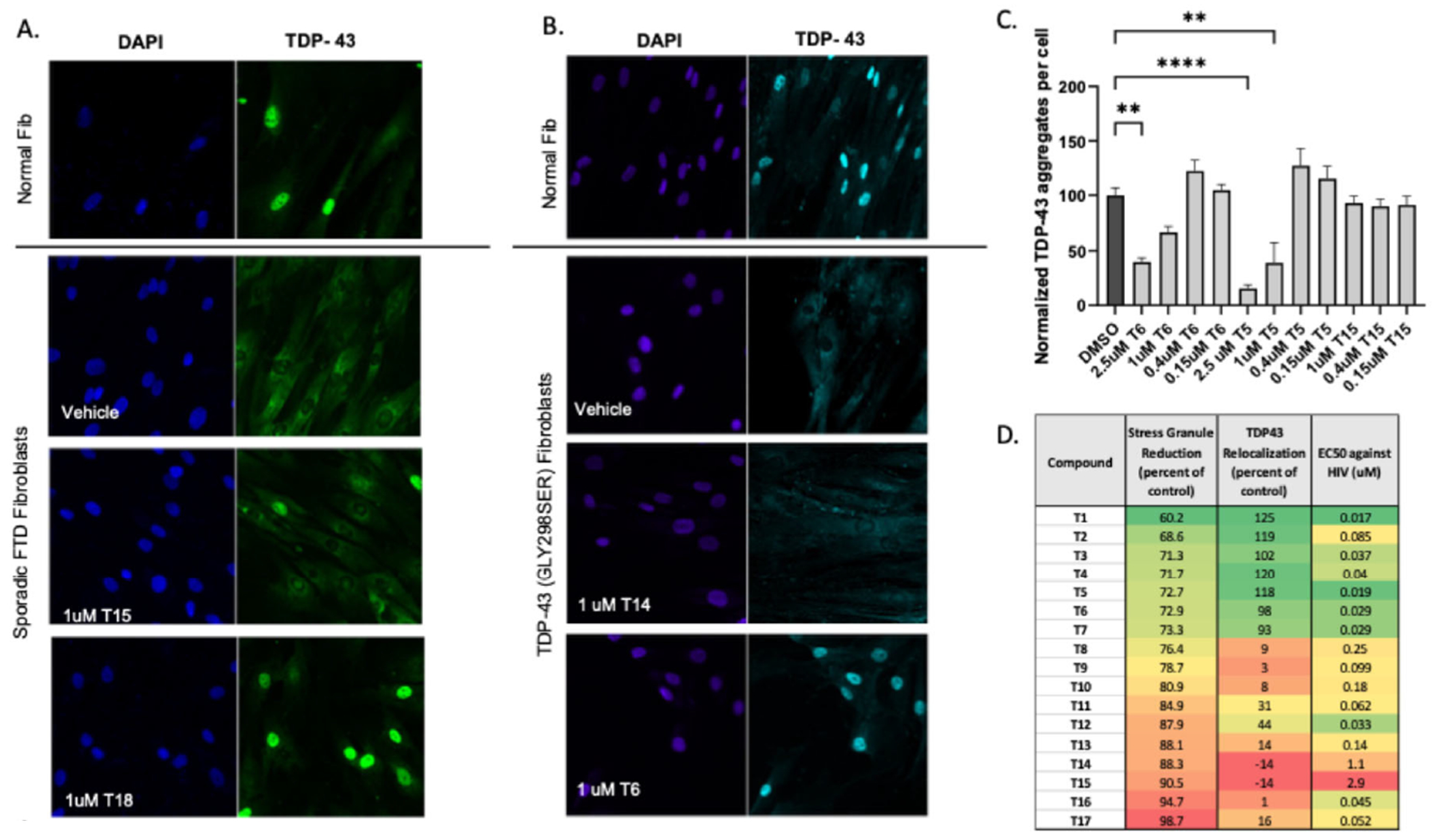
Activity of THIQ assembly modulators in cellular disease models. **Figures 1A** and **1B** show TDP-43 mislocalization and relocalization upon treatment with active compounds for PDFs derived from ALS patients and healthy controls. In the nucleocytoplasmic relocalization assay, cells were seeded and treated with vehicle or 1uM compound for 4 days then washed, fixed, permeabilized and immunostained for TDP-43 and DAPI. The signal intensity of TDP-43 present in the nucleus and cytoplasm of each cell was quantified using CellProfiler software. DAPI images were used to count the total number of cells. Nuc/Cyt ratio was calculated by dividing the intensity of TDP-43 in the nucleus over the intensity of TDP-43 in the cytoplasm of each cell. Nuc/Cyt ratio of the control cells (DMSO-treated cells) was normalized to 100% in each experiment. Compounds capable of relocalizing TDP-43 back to the nucleus show an increase in Nuc/Cyt ratio compared to control cells. **Figure 1C** shows quantitation of stress granule reduction in PDFs following treatment with compound. In the stress granule reduction assay, PDFs were dosed with titrations of 2.5 uM, 1 uM, 0.4 uM, or 0.15 uM compound or vehicle for 24 hours then treated with 500 uM sodium arsenite for one hour. Arsenite was then washed off and cells were fixed, permeabilized, and immunostained for TDP-43, HuR, and DAPI. Cell profiler imaging was used to calculate the number of TDP-43 positive HuR aggregates per cell under each condition and those values were graphed. The graph shows the mean and SEM of triplicate measurements of TDP-43 positive HuR aggregates per cell, normalized to DMSO control values to show relative changes in TDP-43 aggregation. 8 cells per image with 30-50 images were taken so >200 cells for each determination. Statistical significance determined by one-way ANOVA and Dunnett’s multiple comparisons test. **Figure 1D** shows side-by-side comparison of values from the quantitation of the stress granule reduction assay, the nucleocytoplasmic relocalization assay, and activity against infectious HIV for chemical analogs within the THIQ series. In the infectious virus assay, MT-2 cells were infected with NL4-3 Rluc HIV and treated with titrations of 3.3uM, 1.1uM, 0.37uM, or 0.12uM compound or vehicle for four days. Anti-viral activity is shown as the calculated EC_50_.

Fibroblast cells from patients with familial ALS mutations and from sporadic frontotemporal dementia (FTD) patients, as well as healthy controls, were immunostained for TDP-43 [48]. With high content imaging of these immunostained patient-derived fibroblasts (PDFs), the mislocalization of TDP-43 from the nucleus to the cytoplasm is visible in sporadic, TDP-43 mutant, and VCP mutant fibroblasts, but not the healthy fibroblasts (Figures 1A and 1B). The cytosolic TDP-43 could be observed relocalized to the nucleus upon treatment with active THIQ compounds (Figures 1A and 1B). This assay was termed the nucleo-cytoplasmic relocalization assay (NCA).

As a second cellular model for ALS, when PDFs were treated with 500 uM sodium arsenite for one hour and immunostained, cytoplasmic TDP-43 was found co-localized in stress granules along with HuR (Supplemental Figure 2). Treatment with compounds active in the nucleocytoplasmic assay also eliminated the stress granule-associated TDP-43 aggregates in a dose-dependent manner (Figure 1C). This was termed the stress granule assay (SGA). Following the SAR, most active compounds from the nucleocytoplasmic localization assay also displayed similar activity in the stress-induced stress granule assay (Figure 1D).

It occurred to us that the forementioned two cellular assays could be useful for addressing the heterogeneity observed in sporadic ALS patients. The readout of NCA and SGA may correlate to defects that arise from different steps in the complex pathogenesis of ALS. The manifestation of ALS in one sporadic ALS patient may require treatment with a drug that works in NCA. Meanwhile a patient with a different presentation of disease may require treatment with a drug that works in SGA. A compound that shows activity in both assays would be an ideal developmental candidate as it may be able to address symptoms of disease across a heterogenous patient population. In Supplementary Figure 3, we demonstrate this directly by analysis of fibroblasts from three sporadic ALS patients (#11, 13, 14) by NCA and by SGA. As can be seen, some patients show greater defect by NCA as typified by patient #13, others show little NCA defect but a robust defect by SGA, as observed for patient #11. Still others show both kinds of defects (e.g. patient #14). Importantly, the protein assembly modulators assessed here appear to be efficacious on both assays (Supplementary Figure 3).

The structure activity relationship (SAR) of early compounds from the THIQ series in the nucleocytoplasmic relocalization assay in sporadic FTD fibroblasts generally correlated to their activity against infectious HIV in MT-2 cells (Figure 1D). However, the ALS and HIV activities were separable with further medicinal chemistry advancement, where some analogs (ex. compound T16) showed no TDP-43 relocalization but retained activity against HIV in the nanomolar range, while others (ex. compound T2) showed strong TDP-43 relocalization, but substantially weaker antiviral activity compared to other potent compounds (Figure 1D). Similarly, progression of the THIQ lead series resulted in a moderation of toxicity (Supplemental Figure 4). This correlation of lowered toxicity to disease-associated target selectivity is what has been seen previously for the virally modified multi-protein complex in the case of lead series advancement for a structurally unrelated respiratory viral capsid assembly modulator [48] suggesting that the advanced drug is disease-selective and that this represents a general property of advanced assembly modulator small molecules.

### Activity of assembly modulating compounds against animal models of ALS

**Figure 2.**
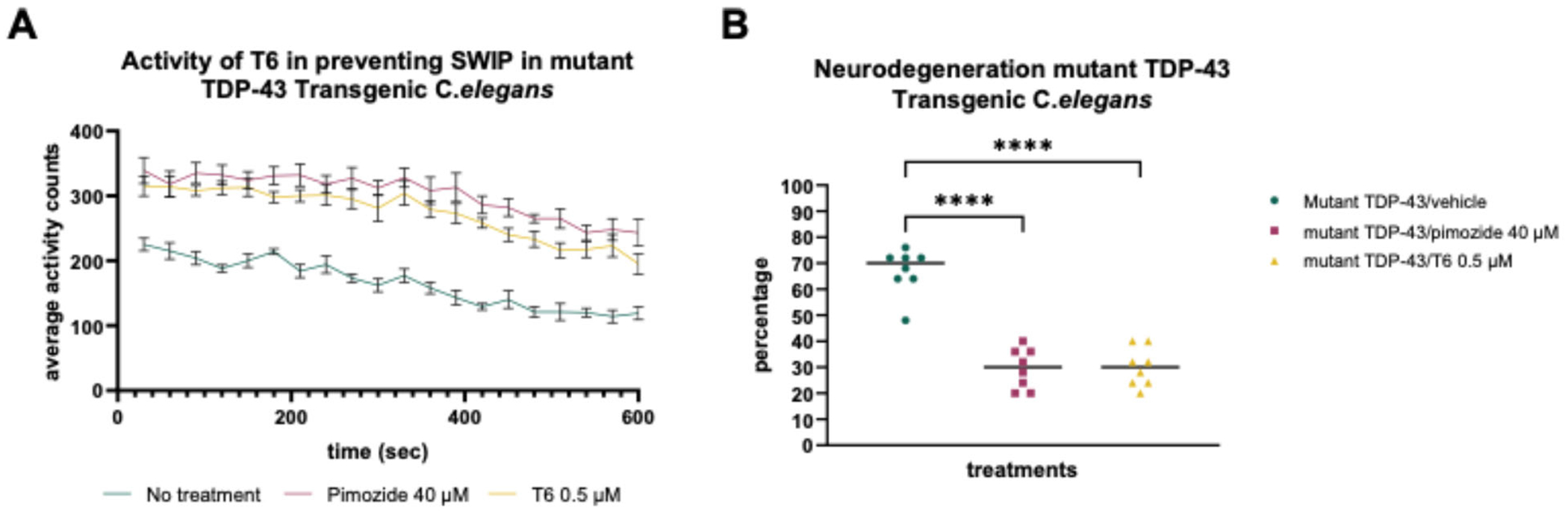
Activity in the *C. elegans* model for ALS. **Figure 2A** shows rescue of *C. elegans* transgenic for the TDP-43 A315T mutation in the SWIP assay. Worms were grown in the presence of 0.5 uM THIQ compound T6, 40 uM pimozide, or nothing and movement was recorded by video. The experiment was performed in 6 replicates of 25 worms each and the average number of body bends per minute is shown for each condition. Simple main effect analysis showed that treatment had a statistically significant effect on average activity counts (F(2,300)=571.4, p<0.0001). **Figure 2B** shows rescue of *C. elegans* transgenic for the TDP-43 A315T mutation in the long term neurodegeneration assay. Worms were grown in the presence of 0.5 uM THIQ compound T6, 40 uM pimozide, or vehicle for 9 days and analyzed for motor neuron splits. Percentage of neurons showing splits is shown for each condition. 8 wells were used in each condition. Asterisks on the graph represent statistical significance was calculated by one-way ANOVA and Tukey’s multiple comparisons test.

After achieving efficacy in multiple cellular models for familial and sporadic ALS, we turned to animal models. Lead compounds of the THIQ series were assessed in *C. elegans* with transgenic human ALS TDP-43 A315T mutation. In the swimming induced paralysis (SWIP) assay, transgenic worms were placed in liquid media containing vehicle or compound at a particular concentration. Worms in the liquid media were scored as “paralyzed” if their body cannot make a bending “S” movement. Efficacy was measured as average body bends per second in vehicle-treated versus compound-treated populations. In the neurodegeneration assay, transgenic worms were grown for 9 days and analyzed for motor neuron splits in the presence of vehicle or compound [58,24]. THIQ compounds demonstrated significant reduction of SWIP and long-term neurodegeneration (Figure 2).

**Figure 3.**
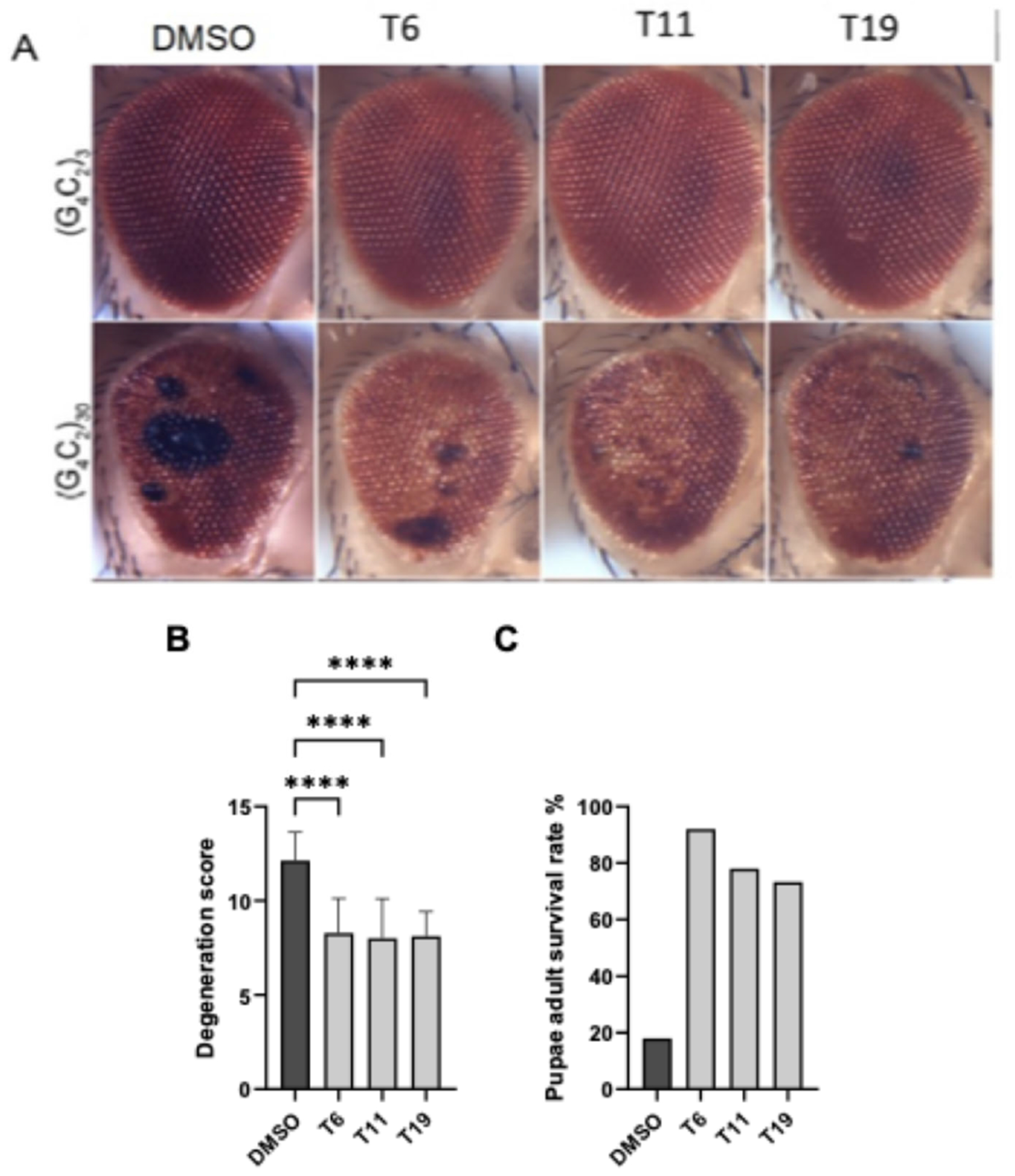
Activity in the *D. melanogaster* model for ALS. THIQ compounds T6, T11, and T12 were tested alongside vehicle in wildtype and transgenic *D. melanogaster* overexpressing C9orf72 30 G4C2 [82,83]. **Figure 3A** shows images of degeneration (black spots) in drosophila eye for wildtype (top row) and transgenic (bottom row) animals. **Figure 3B** shows the corresponding quantitation for degeneration observed in drosophila eye. Shown on graph is the mean and SD from 25 flies. Statistical significance was calculated using one-way ANOVA. **Figure 3C** shows percent adult survival in vehicle versus compound treated conditions.

Active compounds from the series were then assessed in *D. melanogaster* transgenic for the C9orf72 30 G4C2 repeat expansion. Overexpression of 30 G4C2-repeats in fly motor neurons using OK371-GAL4 causes lethality due to paralysis, preventing the exclusion of the adult from the pupal case. Treatment significantly reduced lethality and degeneration caused by 30 G4C2 repeats than when compared with DMSO vehicle alone (Figure 3).

**Figure 4.**
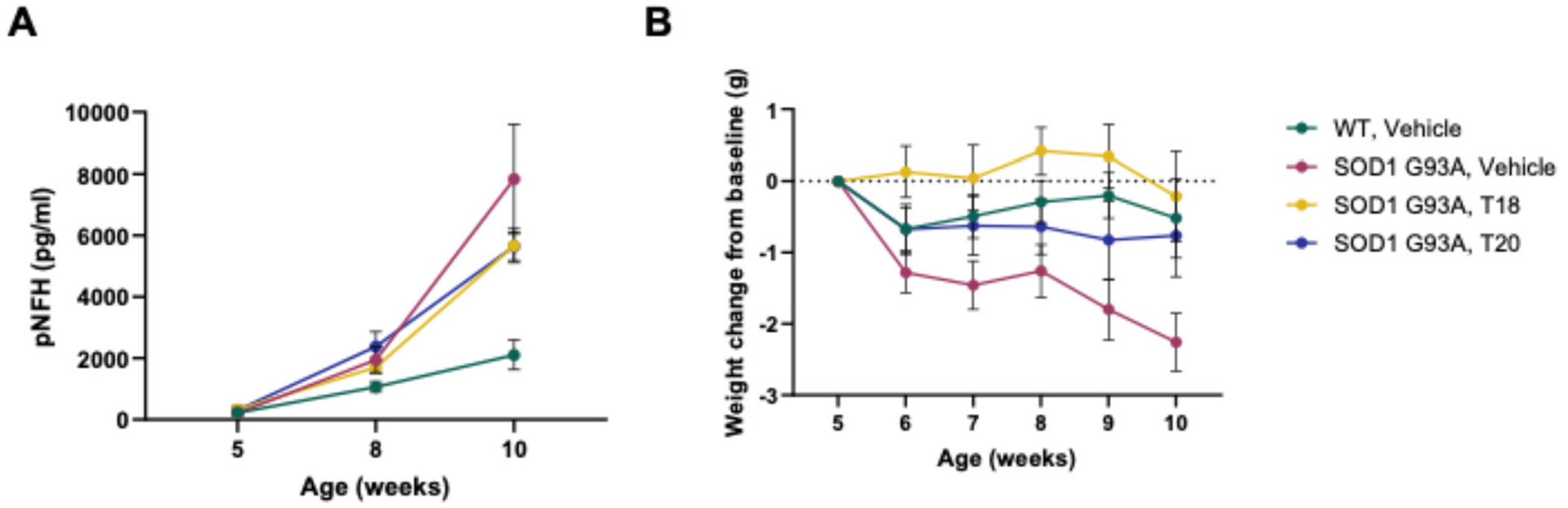
Effect of treatment on pNFH serum levels and body weight of SOD1 mice. Vehicle or compound was administered to mice transgenic for the SODG93A mutation every day for 5 weeks (beginning when the mice were 5 weeks old) by intraperitoneal (IP) dosing. Vehicle was also administered by IP to wildtype mice as a control. **Figure 4A** shows phosphorylated neurofilament heavy chain (pNFH) subunit measured in plasma of the survivors-only mice. Mean and SEM are plotted. Simple main effects analysis showed that treatment had a statistically significant effect on pNFH values (F(3,90)=9.109, p<0.0001). THIQ compounds T18 and T20 were tested alongside vehicle in transgenic mice expressing the SOD G93A mutation. **Figure 4B** shows mean weight change relative to baseline weight taken at week 5 over the course of the study with significant improvements in the compound-treated animals. Error bars represent standard error of the mean. Each treatment group had 12 animals, all females. Simple main effects analysis showed that treatment group had a statistically significant effect on weight change from baseline (F(3,204)=16.55, p<0.0001).

We then wanted to assess activity in a mouse model. First, various compounds in this lead series were shown to be safe in mice, with no effect on weight, behavior, or clinical chemistry or hematology and to have good brain exposure (Supplemental Figures 5 and 6). Two of these safe analogs, T18 and T20, were chosen for assessment in the SOD G93A transgenic (Tg) mouse model of ALS. Vehicle (10% DMSO, 45% propylene glycol, 45% water) or compound was administered to mice Tg for the SODG93A mutation every day for 5 weeks (beginning when the mice were 5 weeks old) by intraperitoneal (IP) dosing [59]. Vehicle was also administered by IP to wildtype mice as a control. Both test compounds showed significant, positive results based on biochemical data (levels of phosphorylated neurofilament heavy chain subunit measured in plasma) and clinical criteria (prevention of weight loss) (Figure 4).

### Target identification in ALS and FTD patient fibroblasts and mouse brain samples

Previous studies on protein assembly modulator small molecule mechanism of action has shown that they target dynamic multi-protein complexes, a feature which appears shared by structurally-unrelated assembly modulators efficacious in disparate therapeutic areas [47,48]. The formation and action of these multi-protein complexes appears to be dependent on metabolic energy (nucleotide triphosphate hydrolysis). Protocols for energy-dependent drug resin affinity chromatography (eDRAC) provided a method to characterize the targets of assembly modulating compounds [47,48]. In those experiments, extract from a disease-relevant cell line or tissue sample are incubated with a modified analog of a compound attached to an Affi-gel resin and serve as an affinity ligand for target identification [60]. The eDRAC experiments made possible tandem mass spectrometry (MS-MS) determination of protein composition of the isolated target multi-protein complexes under various conditions including healthy versus disease cells/tissues, with and without metabolic energy supplementation, and under vehicle versus compound treatment conditions [47,48]. A related method is to couple the drug from the same side group shown irrelevant for biological activity that was used for drug resin construction, instead to a diazirine photocrosslinking moiety and biotin a high affinity ligand selectively removed by its selective binding to streptavidin [61,62]. Isolation under native conditions will reveal the entire multi-protein complex while preceding denaturation allows identification of the direct drug-binding protein. As this approach has been productive for analysis of protein assembly modulators in those therapeutic areas [47,48], we sought to apply the same techniques to the ALS-active assembly modulators presented here, in order to better understand their targets and mechanism.

Cellular extract was prepared from several sources including PDFs, spinal cord tissue of mice that were either wildtype or transgenic expressing the SOD1 G93A ALS-causing mutation, and brain tissue from wildtype vs SOD1 G93A mice in the trial shown in Figure 4, including the cohort treated with NCA and SGA-active THIQ compounds. Extracts were supplemented with an “energy cocktail” of ribonucleotide triphosphates (to a final concentration of 1 mM rATP, 1 mM rGTP, 1 mM rCTP, 1 mM UTP), 4 mM creatine phosphate, and 5 ug/mL creatine kinase. Extracts were incubated with NCA/SGA active THIQ compound coupled to Affi-gel resin or a control resin which consisted of a blocked affi-gel matrix, for an hour at 22°C [33]. The resins were washed with 100 bed volumes of buffer and eluted with 100 uM compounds containing the energy cocktail first for two hours at 22°C, eluate collected and a second elution performed overnight with energy cocktail at 22°C, followed by stripping the column with SDS.

**Figure 5.**
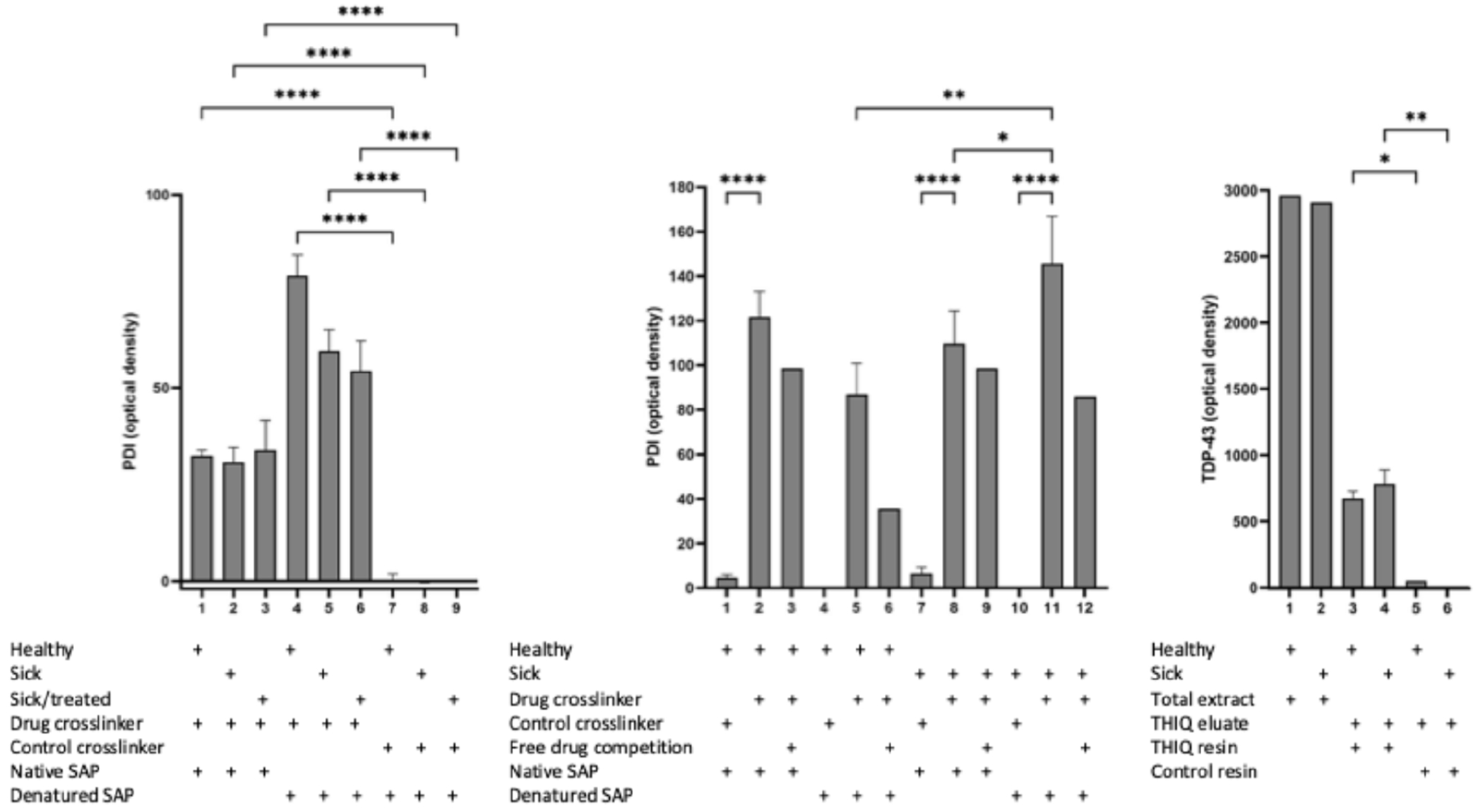
Target identification in mouse brain and PDF samples. **Figure 5A** shows the results of a photocrosslinking study in which healthy (bars 1, 4, 7), sick SODG93A (bars 2, 5, 8) or sick-THIQ-treated (bars 3, 6, 9) mouse brain was assessed with PAV-073 crosslinker (bars 1-6) under native (bars 1-3) or denatured (bars 4-6) conditions. Bars 7-9 show use of control crosslinker eliminates PDI precipitation. Mean of three replicates for each condition and SD are shown on graph. Statistical significance was determined by one-way ANOVA and Šídák’s multiple comparisons test, indicated by asterisks on the graph. In **Figure 5B** photocrosslinking is done with healthy (bars 1-6) versus ALS PDF extracts (bars 7-12) handled in much the same way as in **Figure 5A** (native SAP bars 1-3 and 7-9; denatured bars 4-6 and 10-12), except that an additional control was performed in which crosslinker-biotin lacking the drug was shown not to precipitate PDI (bars 1, 4, 7, 10). Free drug competitor is shown in lanes 3, 6, 9, 12). For both **5A** and **5B** after crosslinking, the samples were divided in two, one of which was denatured in 1% SDS at 100°C for 3 minutes before addition of excess non-denaturing detergent (Triton-X-100) to take up the free SDS into micelles. Streptavidin beads were added and the bound protein was precipitated and analyzed by western blot for PDI. Quantitation of the protein band for PDI is shown as arbitrary density units. Total inputs and selected controls were done in single point. Otherwise, experiments were done in triplicate with bars representing the average density and error bars representing the standard deviation. Statistical significance was determined by one-way ANOVA and Šídák’s multiple comparisons test, indicated by asterisks on the graph. **Figure 5C** shows representative results of mouse brain THIQ and control resin free drug eluates western blotted for TDP-43 (same samples sent for MS-MS). In all cases, arbitrary density units are shown on the y-axis which allows in **Figure 5C** comparison of matched samples of total extract (lanes 1, 2) with THIQ drug resin (lanes 3,4) and control resin (lanes 5, 6). The total shown on the blots is 1/66 of the total applied to each resin. The eluates shown are 1/10 of the free drug eluate generated. Statistical significance was determined by one-way ANOVA and Šídák’s multiple comparisons test, indicated by asterisks on the graph.

**Figure 6.**
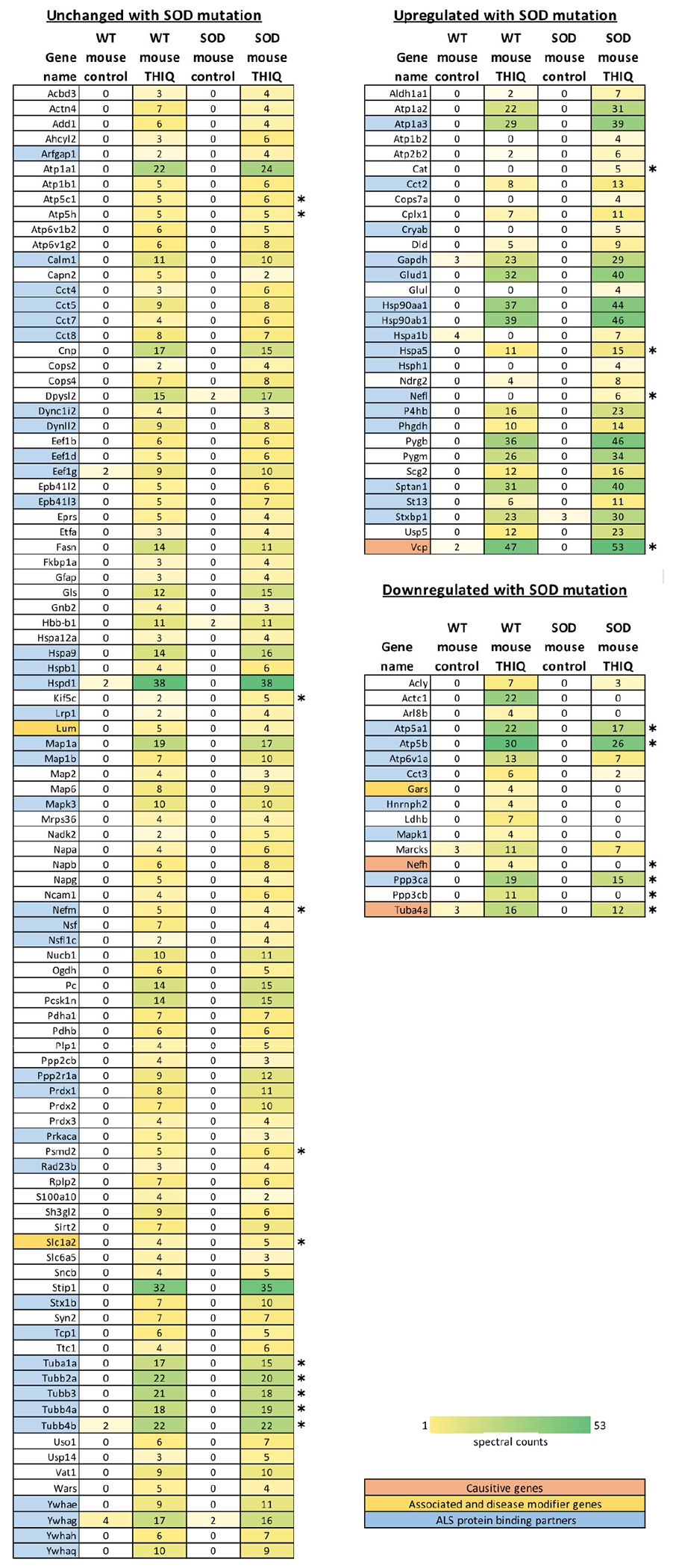
Analysis of wildtype (WTE) and SOD1 G93A mutant mouse brain (MTE) THIQ drug resin eluates. Spectral count values in WTE and MTE with proteins categorized as downregulated, upregulated, and unchanged in MTE, along with their corresponding spectral counts for WTE, and both control eluates. Proteins associated with KEGG’s ALS disease pathway are marked with a “✱” on the right side of the tables. Causative genes, as identified by Dervishi [19], are highlighted in orange, associated and disease modifier genes in yellow, and ALS protein interactors in blue.

The eluate from mouse brain, mouse spinal cord, and patient fibroblasts was characterized by photocrosslinking and western blotting (Figure 5). The eluate from mouse spinal cord was also analyzed by MS-MS (Figure 6).

### Photocrosslinking and western blot studies

To determine the direct drug-binding protein, a photocrosslinker compound was synthesized in which diazirine and biotin moieties were attached at the same position used previously to attach to the resin. Thus, upon exposure to UV light, a covalent bond is formed between the diazirine moiety of the compound and the nearest neighbor protein. Under native conditions (in the presence of metabolic energy) the full complex is isolated. However upon denaturation, followed by streptavidin precipitation (SAP), only the nearest neighbor drug-binding protein, to which an irreversible covalent crosslink has been achieved, is recovered and is identifiable by western blotting. Associated proteins that are not direct drug-binding proteins, will be identified under native conditions, but will be lost upon denaturation.

Compound PAV-073 was chosen as the analog to be used as a photocrosslinker for the THIQ series because its activity is selective to ALS, having largely lost efficacy against infectious HIV (see Supplemental Figure 1B for activity of PAV-073 and Supplemental Figure 7 for synthetic scheme of PAV-073 crosslinker). Crosslinking and SAP from mouse brain extract (brains from the SODG93A efficacy study, including wildtype, transgenic mutant, and transgenic mutant/compound treated animals) identified protein disulphide isomerase (PDI) as a direct drug target. Western blot analysis showed a protein band for PDI present in both native and denatured samples when crosslinked with PAV-073 but not the negative control (Figure 5B). Furthermore, the PDI band was diminished with presaturation, where the free PAV-073 was added to the sample before crosslinking to compete out binding-sites (Figure 5B).

The crosslinking experiment was repeated using cellular extract prepared from PDFs from a sporadic ALS patient (#51) and healthy control (#27) fibroblasts (Figure 5B). For both PDF and brain samples, PDI was present as a target in both healthy and ALS conditions (Figures 5A and 5B).

Additional Western blots were run on wildtype and SOD1 G93A mutant mouse spinal cord samples that were also analyzed by mass spectrometry. TDP-43 was identified by western blot in both THIQ drug resin eluates but not in control resin eluates (Figure 5C). Notably, the TDP-43 bound to the drug resin was a small fraction (6%) of the total TDP-43 in the extract, consistent with the hypothesis that only small subsets of the total amount of individual proteins present in a cell are components of this novel, transient and energy-dependent drug target.

### Mass spectrometry analysis of THIQ eluates

The overnight eluates of wildtype mouse and SOD1 G93A mouse spinal cord eDRAC eluates from the THIQ drug resin were analyzed by MS-MS. To eliminate non-specifically bound proteins from the MS-MS dataset, we removed proteins with less than 4 spectral counts in the THIQ eluate and those with more than 4 spectral counts in the control eluates. In cases where a protein had more than 4 spectral counts in one THIQ eluate but not the other, we retained the sub-threshold spectral count value for comparison. After this background subtraction, we identified 138 proteins in the WT THIQ eluate (WTE) and 136 proteins in the SOD1 G93A mutant THIQ eluate (MTE) by these criteria. Of these, 128 proteins were found in both WTE and MTE, while 10 proteins were unique to WTE and 12 were unique to MTE (Figure 6).

Although most proteins were shared between WTE and MTE and had comparable spectral count values, small subsets of proteins were enriched or depleted in MTE. Specifically, 31 proteins had a 4 spectral count or higher increase compared to WTE, 17 proteins had a 4 spectral count or more decrease compared to WTE, and 98 proteins remained relatively unchanged (Figure 6). Comparable single digit percent of total extract apply to PDI and other proteins in the free drug eluate samples analyzed by MS-MS here, as described for Figure 5C.

### ALS interactome analysis of THIQ eluates

Several databases were interrogated to determine the relevance of proteins found in the THIQ eluates concerning ALS. Specifically, the KEGG database consists of genes whose encoded proteins comprise pathways that are perturbed in disease states [63]. The Derevishi “ALS proteins” list comprises the 147 mutations that have been identified in ALS patients [19] (of these, 39 are widely accepted as causative and 108 are accepted as associated or as modifying disease initiation or progression) and 1105 experimentally proven, high confidence protein binding partners are also included. DAVID (Database for Annotation, Visualization, and Integrated Discovery) is a bioinformatics resource that preforms function annotation clustering of biological functions with redundant or similar gene products [64].

To explore the involvement of proteins in the THIQ eluate in ALS, we conducted a KEGG pathway analysis using DAVID (Supplemental Figure 8). Among the 145 proteins from the THIQ eluates, 21 were annotated by KEGG as part of the ALS disease pathway map (Figure 6A and 6C). These proteins were associated with four sub-pathways: oxidative phosphorylation (ATP5F1A, ATP5F1B, ATP5F1C, ATP5PD), regulation of actin cytoskeleton (CAT, KIF5C, NEFH, NEFL, NEFM, TUBA1A, TUBA4A, TUBB2A, TUBB3, TUBB4I), proteasome (HSPA5, PPP3CA, PPP3CB, PSMD2, VCP), and glutamatergic signaling (SLC1A2).

Additionally, we compared our proteins to the Dervishi “ALS proteins” database [19]. Among the 145 proteins identified in the THIQ eluates, 66 were ALS proteins (46%), indicating a strong enrichment of ALS-related proteins in our eluate (Figure 6). Notably, we found 3 causative proteins (VCP, TUBA4A, and NEFH), 3 disease modifier proteins (SCL1A2, LUM, and GARS), and 60 ALS protein binding partners, highlighting their potential relevance in ALS pathophysiology.

### Gene ontology biological processes analysis of THIQ eluates

To elucidate the biological functions of the proteins in the THIQ eluates, we conducted a Gene Ontology (GO) biological process analysis using DAVID [64]. The analysis revealed seven enriched clusters of biological processes: protein folding, ion regulation, ATP synthesis, vesicle transport, protein localization, redox, and protein neddylation/deneddylation (Supplemental Figure 9A).

Furthermore, we performed a separate Gene Ontology analysis of biological processes for proteins that were downregulated, upregulated, and unchanged in MTE. This analysis unveiled distinct patterns: upregulated proteins were enriched in protein folding and ion regulation pathways, downregulated proteins enriched in energy generation (ATP synthesis). Proteins whose abundance remained unchanged proteins in comparing the MTE to the WTE included protein folding, microtubule organization, protein localization, and redox (Supplemental Figure 9B).

## Discussion

### Protein assembly modulators display unprecedented activity in models for ALS

Activity of compounds in the THIQ protein assembly modulator chemical series appear to normalize an array of hallmarks of ALS pathology including elimination of stress-induced TDP-43 aggregates in stress granules, rescue of TDP-43 mis-localization (Figure 1), reversal of paralysis in worms (Figure 2), reversal of neurodegenerative markers in all models (Figures 2–4), normalization of weight in mice (Figure 4), and increased lifespan in worms and flies (Figures 2 and 3). This chemotype appears to work for both familial and sporadic forms of ALS. Efficacy in sporadic ALS is supported by data from NCA or SGA assays in sporadic ALS PDFs. Efficacy in familial ALS is supported by the data in cellular assays as well as in transgenic *C. elegans* (worms), *D. melanogaster* (flies) and mice bearing a diversity of ALS-causing transgenes (TDP-43 A315T, c9orf72 repeat expansion, and SODG93A, respectively (Figures 1–4). This is consistent with the expectation that protein assembly modulation serves to correct dysregulation at any of several steps in the pathophysiological cascade resulting in ALS. Therefore, assembly modulation may be therapeutic for a wide range of ALS-causing defects, upstream of differences between sporadic and familial ALS.

### Protein assembly as a druggable process, in context

The premise in support of our unconventional approach to drug discovery and our pivot from focusing on viral to nonviral diseases, once hit compounds were identified and suitably advanced, was that viruses have used deep evolutionary time and natural selection to find the most efficient ways to take over our cells and prevent activation of host defensive measures. The targets the viruses discovered likely represent weak links of human biology at risk for diseases involving departures from homeostasis-- including those not caused by viruses.

We do not suggest that viruses cause neurodegenerative diseases, including ALS. Instead, we propose that the shared consequence of both viral infections and the non-viral diseases is disruption of homeostasis. This occurs by a specific molecular mechanism that involves critical components involved in protein assembly. These components can be manipulated with allosteric site-targeted protein assembly modulator small molecule drugs, to therapeutic advantage, as demonstrated here.

Given that the natural history of acute viral infection unfolds over hours while that of neurodegeneration is believed to progress over years to decades, it is not surprising that these compounds were first discovered for their anti-viral efficacy. It is perhaps also not surprising that a method such as cell-free protein synthesis, with demonstrated value in revealing biological complexity, should enable detection of these unconventional transient, energy-dependent multi-protein complex targets [42,44]. Likewise it should also not be surprising that viruses would discover a class of targets through natural selection that remain inaccessible to conventional proteomics or genetics, as features of biology not easily parsed by conventional methods appear involved [37,40].

Our use of viral assembly as a surrogate for discovery of small molecule protein assembly modulators of relevant allosteric sites for the restoration of homeostasis has proven productive. Identification of allosteric sites, which affect activity of a small subset of a given gene product, cannot be easily achieved through genetic manipulation, due in part to the confounding effects of protein “moonlighting” [65,66]. Of necessity, genetic manipulation affects all of the diverse forms of a particular gene product, irrespective of their different functional roles in physiology. Since only one of these functions may be responsible for the disease, and others may be protective against the disease, targeting all forms and all functions, may obscure therapeutic effect. Finding a small molecule that selectively targets only the relevant subset of protein-protein interactions provides a path to retain broad activity while avoiding toxicity, as demonstrated for other protein assembly modulator compounds [47,48], and suggested for ALS from the data presented here.

### Protein assembly modulators target multi-protein complexes

For multiple areas of disease, and through multiple classes of chemical compounds, protein assembly modulators target dynamic multi-protein complexes via various proteins implicated in allosteric regulation. One chemotype series active against all respiratory viruses accomplishes this by targeting 14-3-3 proteins [48,67]. Another chemotype active against all 80 cancers studied targets a multi-protein complex that includes KAP1/TRIM28 [47,68]. The protein assembly modulator active in the Alzheimer’s Disease mouse, targets MIF [49]. The ALS/HIV active chemotype described in this paper targets PDI, a protein also implicated in allosteric regulation and in cleavage of disulfide bonds [69–72]. Variants of PDI with single nucleotide polymorphisms and redistribution of PDI within different regions of the cell, including endoplasmic reticulum sub-compartments, are implicated in the literature as correlating with ALS symptoms [73–75]. Furthermore, overexpression of PDI has been observed to protect mice against neurodegeneration in the SOD1G93A model as well as multiple cellular models of ALS [74,76–78]. The PDI detected in the compound’s target by eDRAC of both healthy and ALS patient fibroblast cells, appears to be interacting with other proteins in a similarly energy-dependent manner as previously described for the other assembly modulator compounds shown efficacious in other therapeutic areas [47,48].

The MS-MS data analysis of eDRAC eluates provides valuable insights into the molecular pathways targeted by our drug. Typically, proteomic experiments are conducted using whole cell extracts, where changes in protein abundance are measured in response to various experimental manipulations. However, our study takes a more focused approach, isolating the proteins specifically bound to our drug using eDRAC methods. Through KEGG pathway analysis, we identified our drug target in several pathways, including oxidative phosphorylation, regulation of actin cytoskeleton, the proteasome, and glutamatergic signaling. Comparison with the Dervishi *et al.* database revealed almost half of the proteins in our target are part of pathways that converge on an ALS phenotype [19]. The Gene Ontology analysis revealed seven distinct and highly enriched clusters of biological processes in which proteins present in our target are involved (supplemental Figure 7) [79].

The fact that the 145 proteins found in our drug target resisted extensive washing before elution from the drug resin by free drug, suggests that they form a network of protein-protein interactions. In essence, these proteins are interconnected and likely participate in cooperative cellular processes. While the precise roles of the small subsets of these proteins bound to the drug resin in both healthy and diseased cells remain unknown, their collective influence on ALS disease pathology is evident from the data provided here.

This approach to drug target identification offers some notable advantages over conventional approaches. First, as it starts with a phenotypically active drug, it provides a window into a biologically robust but poorly understood dimension of ALS pathophysiology. Second it reveals that the interconnectedness and multifunctionality of protein complexes within cells is relevant to ALS. Although the target proteins appear to be involved in various distinct and seemingly unrelated pathways, we show small subsets of the identified proteins to be physically together in a multi-protein complex that is druggable. As with the targets of the other assembly modulators, the protein composition of the multiprotein complex ALS drug target differs between healthy and sick cells (Figure 6). Moreover, the efficacy of our drug against ALS in various models and species (worm, fly, mouse, human cells) suggests that the biological processes or functions targeted by our drug are conserved to some extent across these diverse organisms, a theme highlighted previously by others [80].

TDP-43 was detected by western blot in both wild type and SOD1 G93A mutant mouse spinal cord drug resin eluates and not in control resin, consistent with the demonstration of drug efficacy at correction of TDP-43 mislocalization. Only a small fraction of total cellular TDP-43 is accounted for by what binds to the drug resin. This is consistent with findings with other assembly modulators that demonstrate only a small fraction of the total of component proteins in the cell are actually in a specific multi-protein complex target. Possibly the biological activity of interest represents a “moonlighting” function [66] of that small subset of the relevant proteins. Alternatively, the small subset of TDP-43 bound to the drug target multi-protein complex may be a steady state intermediate, e.g. in nucleocytoplasmic transport.

We hypothesize that the unique properties observed for protein assembly modulators may be attributable to their having a mechanism of action that works via allosteric regulation. This dimension of biological regulation remains poorly understood [81] but is likely highly relevant to re-establishment of homeostasis. Targeting allosteric sites instead of active sites may be an effective strategy to selectively inhibit activity of the subsets of proteins which have been dysregulated in a diseased state, without targeting those copies of the same protein which remain functional, including for unrelated functions that are not affected by ALS pathophysiology. Modulation of multi-protein complexes that catalyzes protein assembly may provide a means of restoring homeostasis, rather than simply blocking a disease-associated form, which alone does not restore homeostasis.

### Significance of the findings

**Figure 7.**
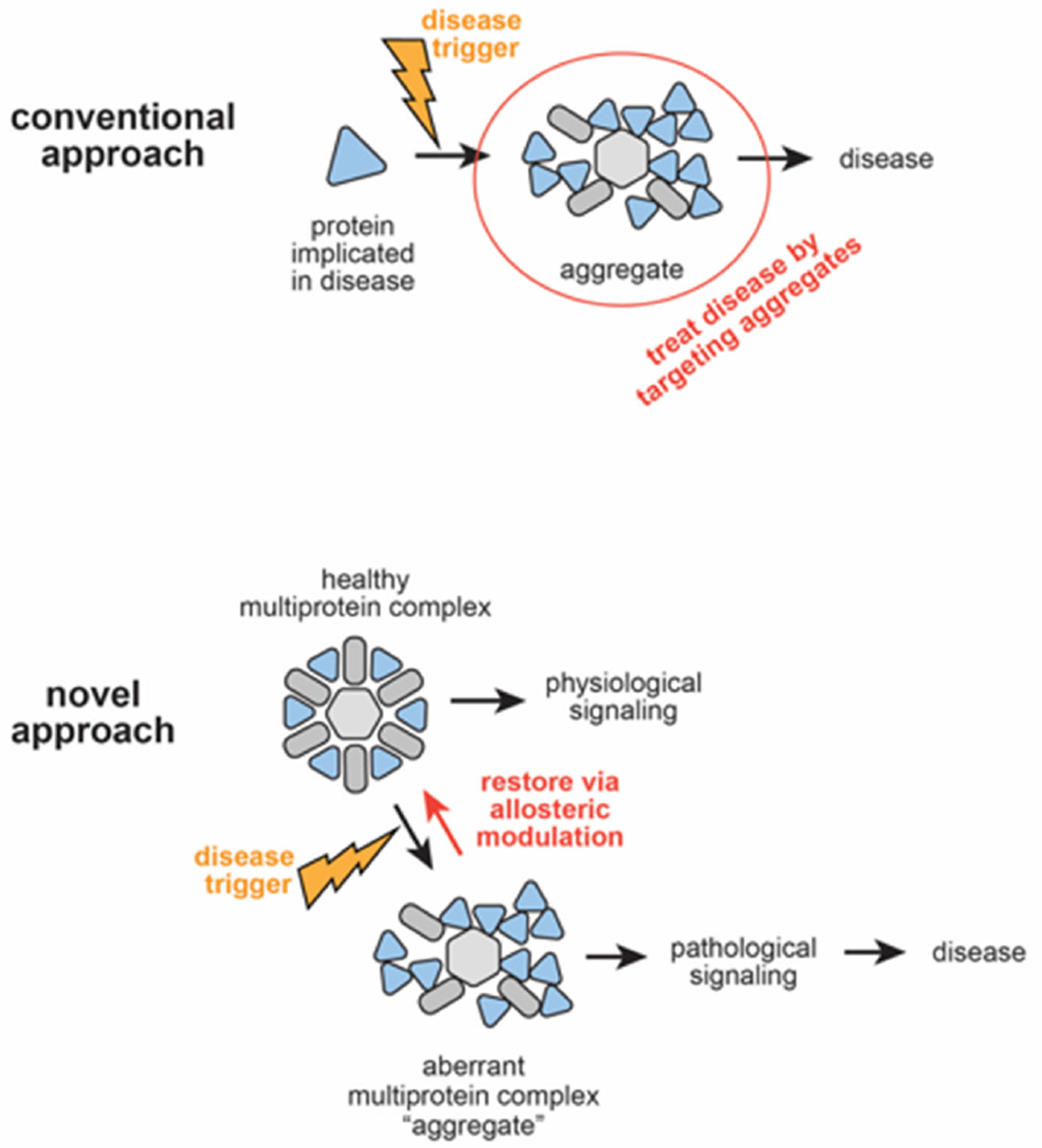
Protein assembly modulation drug action. Hypothesized mechanism of protein assembly modulator drug action for protein aggregation diseases including ALS, based on the data. Top: conventional approaches aimed at removal of aggregates. Bottom: Protein assembly modulation approach to preventing aggregate formation. By targeting an allosteric site to prevent aggregate formation, rather than targeting aggregates after they have formed, the outcome is akin to installing a stop sign to prevent accidents at a busy intersection, rather than sending tow trucks to remove wreckage once an accident has occurred.

Efficacy of the same protein assembly modulator small molecule in both familial and sporadic ALS supports the hypothesis that these compounds act on an upstream regulatory mechanism rather than on downstream consequences of disease (manifest as protein aggregates). Thus one therapeutic small molecule is broadly applicable despite the heterogeneity of ALS. As an analogy, when car crashes occur at a busy intersection with a broken traffic light, upstream preventative solutions, like installing a stop sign or fixing the broken traffic light, are more effective than a focus on downstream consequences of dysregulation, such as sending more tow trucks to remove roadside wreckage. Sending tow trucks to remove the wreckage will improve traffic in the short term, but if the underlying defect which led to the crash is not addressed— it is only a matter of time before it happens again. Targeting the aggregates themselves is akin to eliminating wreckage solely by sending tow trucks following a crash. Allosteric modulation on the other hand, is a way to prevent wreckage by fixing the broken traffic light [82]. Figure 7 illustrates the distinction between conventional thinking with regards to protein aggregate-associated diseases including ALS, and the hypotheses formulated and tested here.

Another implication of our findings is that, though the targets of the viral and related non-viral diseases are similar, they are nevertheless sufficiently distinctive that their activity can be separated through SAR advancement. We observed early compounds in the THIQ series show potency for both ALS and HIV. However, the advanced ALS-active compound PAV-073, demonstrated increased potency in the worm model for ALS while showing substantial loss of activity against infectious HIV in cell culture. Not shown, a different advanced subseries of this chemotype is low double digit nanomolar active against HIV, but without activity in the worm ALS model. This suggests that there are two separate targets (one relevant for ALS, one for HIV) for this chemical series. This unexpected feature emerging from the SAR has an important implication for ALS therapeutics. Driving the SAR towards selectivity for ALS will likely diminish liability for off-target toxicity and illuminate the molecular mechanisms underlying each disease state in ways that have been heretofore inaccessible.

Finally, what is particularly distinctive about our approach is the use of viruses as “trufflehounds” [35] to reveal the otherwise hard to detect allosteric sites by which this novel dimension of biological regulation occurs, applied here to ALS therapeutics.

## Conclusion

ALS is a devastating disease and novel treatments are urgently needed. Our orthogonal approach to drug discovery has led to a novel target and mechanism with remarkable efficacy at rescue of both TDP-43 mislocalization and stress-induced aggregation, the biochemical hallmarks of ALS, with improvement of symptoms observed in a diversity of transgenic animal models for ALS. We hypothesize this mechanism to reflect a molecular basis for homeostasis that is disordered in ALS and for which these novel drugs appear therapeutic. This conclusion is demonstrated here in a diversity of cellular and animal models of ALS and is corroborated by MS-MS analysis of the drug resin free drug eluate revealing an enrichment of proteins of the ALS interactome.

## Materials and Methods

### Lead contact and materials availability

Further information and requests for resources and reagents should be directed to and will be fulfilled by the Lead Contact Vishwanath R. Lingappa (vlingappa@prosetta.com).

Use of unique compound PAV-073 and its stable derivatives may be available upon request by the Lead Contact if sought for experimental purposes under a valid completed Materials Transfer Agreement.

### DRYAD accession links:

#### Reviewer share link:

https://datadryad.org/stash/share/9P-_-rZqCJY14ZmEb20D8zavYhLiuzeKwFo4mMxn-rI

Chemical Synthesis (Supplemental Figures 1 and 3)

#### Synthesis of PAV-073

A mixture of 3-hydroxy-4-methoxyacetophenone (16.6 g, 100 mmol), benzyl chloride (13.8 mL,120 mmol), and anhydrous K_2_CO_3_ (20.7 g, 150 mmol) in DMF (100 mL) was heated at refluxed for 5 h. The reaction mixture was concentrated to dryness, the residue was redissolved in EtOAc (100 mL) and then washed with 5% aqueous NaOH (3x30 mL). The organic layer was washed with brine (2x10 mL) and H_2_O (2x30 mL), dried (Na_2_SO_4_) and evaporated to a residue, which was purified by flash chromatography to provide 1 (22.9 g, 90%). MS (m/z):257 [M+H].

NaH (60 wt % in mineral oil, 1.95 g, 48.5mmol) was suspended in THF (100 mL) and cooled to 0°C. Triethyl phosphonoacetate (9.6 mL, 48.5 mmol) was added dropwise and the reaction mixture was stirred at 0°C for 30 min. Then 3-benzyloxy-4-methoxy-acetophenone (1) (6.2 g, 24.2 mmol) was dissolved in THF (0.1 ml/mmol) and added to the reaction mixture. The cooling bath was removed, and the mixture was stirred at 50°C until full conversion was detected (TLC). The reaction mixture was quenched by slow addition of H_2_O (2 ml/mmol ketone), extracted with t-butyl methyl ether (3x3 ml/mmol) and the combined organic layers were dried (Na_2_SO_4_) and evaporated to give a residue, which was purified by flash column chromatography to provide compound 2 (6.4 g, 81%). MS (m/z): 327 [M+H].

A mixture of ethyl ester (2) (6.4 g, 19.5 mmol) and alcoholic potassium hydroxide (4.0 g, 71 mmol KOH/ 100 mL EtOH) was stirred at room temperature for 12 h. The solution was then concentrated to give a residue, which was purified by flash column chromatography on silica gel to provide 3 (5.6 g, 96%). MS (m/z): 299 [M+H].

A suspension of 3-(3-benzyloxy-4-methoxyphenyl)-2-butenoic acid (3) (5.6 g, 18.8 mmol), CuBr (270 mg, 1.9 mmol) and tertiary butyl nitrite (8.9 mL, 37.6 mmol) in acetonitrile (50 mL) was stirred at 80°C for 18 h. Reaction completion was monitored by TLC. After completion, the reaction mixture was cooled to room temperature, solvent was removed under reduced pressure and the crude product was purified by flash chromatography to yield compound 4 (3.9 g, 70%). MS (m/z): 300 [M+H].

To a solution of 3-(3-benzyloxy-4-methoxyphenyl)-l-nitro-2-butene (4) (3.9 g, 13.2 mmol) in 40 mL of anhydrous THF under argon was slowly added a 2.0 M solution of LiAlH_4_ in THF (40 mL, 80 mmol) and the reaction mixture was heated at refluxed for 2 h. The reaction mixture was cooled and excess reagent was quenched by dropwise addition of H_2_O and 15% aqueous NaOH. The reaction mixture was extracted with CH_2_Cl_2_ (3x30 mL) and the combined organic layers were treated with 5% aqueous HCl. The aqueous acid layer was then basified (5% aqueous NH_4_OH, pH 9) and extracted with CH_2_Cl_2_. The organic solution was washed with brine (2x30 mL) and H_2_O (2x30 mL), dried (Na_2_SO_4_) and evaporated to give compound (5) (2.3 g, 63%). MS (m/z): 272 [M+H].

To the stirred solution of (E)-3-(6-methyl-l,3-benzodioxol-5-yl)prop-2-enoic acid (8) [see synthesis below] (140 mg, 0.68 mmol) and 2-(3-benzyloxy-4-methoxy-phenyl)propylamine (5) (185 mg, 0.68 mmol) in DMF (2 mL) was added HATU (310 mg, 0.82 mmol) and diisopropylethylamine (351 mg, 0.473 mL, 15.0 mmol). The reaction mixture was stirred at room temperature for 1 h, diluted with EtOAc (50 mL), washed with 10% citric acid, saturated aqueous solution of NaHCO_3_, dried (Na_2_SO_4_), filtered and evaporated to give a residue, which was purified by flash chromatography (ethyl acetate/hexanes) to provide compound 6. Yield 11.4 mg (35% overall yield from nitrostyrene). MS (m/z): 460 [M+H].

A suspension of (E)-N-[2-(3-benzyloxy-4-methoxy-phenyl)-propyl]-3-(6-methyl-l,3-benzodioxol-5-yl)-prop-2-enamide (6) (110 mg, 0.24 mmol) in dry acetonitrile (10 mL) was heated at reflux. Then phosphorus oxychloride (400 mg, 0.24 mL, 2.6 mmol) was added drop wise and the reaction mixture was heated at reflux for an additional 1 h. The solvent and reagent were evaporated under vacuum, the organic layer was washed with water (2x10 mL). and evaporated in vacuo to give an oil, which was then dissolved in ethanol (8 mL) and sodium borohydride (9.8 mg, 0.26 mmol) was added. The reaction mixture was stirred at room temperature for 30 min and excess reagent was destroyed by dropwise addition of 2 M HC1. The reaction mixture was basified with 2 M NaOH and ethanol was removed in vacuo to give a residue, which was partitioned between water (10 mL) and chloroform (10 mL). The organic layer was washed with water (2x10 mL), dried and evaporated to give a residue, which was purified by column chromatography (dichloromethane/methanol) to give 6-nenzyloxy-7-methoxy-4-methyl-l-[(E)-2-(6-methyll, 3-benzodioxol-5-yl)-vinyl]-1,2,3,4-tetrahydroisoquinoline (7) (10 mg, 10%). MS (m/z): 444 [M+H].

#### Synthesis of PAV-073 photocrosslinker

To 6-(tert-Butoxycarbonylamino)-2-(9H-fluoren-9-ylmethoxy carbonylamino)-hexanoic acid 9 [468mg (1 mmol)] in a 40 ml screw top vial was added 4N HCl in Dioxane (3 ml). The vial was sealed and gently agitated for 20 min at room temperature. The mix was then rotary evaporated to dryness and the residue placed on high vacuum overnight.

The dried residue was taken up into 4 ml of DMF (anhydrous) and then sequentially treated with 3-(3-Methyldiazirin-3-yl)-propanoic acid [128 mg (1 mmol)], and DIEA [695 ul (4 mmol)]. With rapid stirring, under Argon atmosphere, was added dropwise HATU [380 mg (1 mmol)] dissolved in 1 ml of DMF. After stirring for 30 min the mixture was quenched with 10 ml of sat. NH_4_Cl solution and then extracted 2 x with 10 ml of EtOAc.

The combined organic extracts were washed once with sat. NaCl, dried (Mg_2_SO_4_) and then rotary evaporated to dryness. The residue was purified by flash chromatography, using a gradient of Ethyl acetate and Hexane, affording 10 (293 mg) in 61% yield.

To 6-benzyloxy-7-methoxy-4-methyl-1-[(E)-2-(6-methyl-1,3-benzodioxol-5-yl)vinyl]-1,2,3,4-tetrahydroisoquinoline 7 [15 mg (0.03 mmol)] in a 40ml screw top vial was added 4N HCl in Dioxane (0.5 ml). The vial was sealed and gently agitated for 20 min at room temperature. The mix was then rotary evaporated to dryness and the residue placed on high vacuum overnight.

The dried residue was taken up into 1 ml of DMF (anhydrous) and then sequentially treated with 10 [14.5 mg (0.03 mmol)], and DIEA [32 ul (0.18 mmol)]. With rapid stirring, under Argon atmosphere, was added dropwise HATU [14.6 mg (0.038 mmol)] dissolved in 300 ul of DMF. After stirring for 30 min the mixture was quenched with 5 ml of sat. NH_4_Cl solution and then extracted 2 x with 5 ml of EtOAc.

The combined organic extracts were washed once with sat. NaCl, dried (Mg_2_SO_4_) and then rotary evaporated to dryness. The residue was purified by flash chromatography, using a gradient of Ethyl acetate and Hexane, affording 11 (28 mg) in quant. yield.

To 11 [28 mg (0.03 mmol)] in a 40 ml screw top vial was added 50/50 Diethylamine / DMF (0.5 ml). The vial was sealed and gently agitated for 60 min at room temperature. The mix was then rotary evaporated to dryness and the residue placed on high vacuum overnight. The residue was triturated 2 x with 3 ml of Hexane to remove the Dibenzofulvene amine adduct. The residue was again briefly placed on high vacuum to remove traces of Hexane. The dried residue was taken up into 1 ml of DMF (anhydrous) and then treated with Biotin-PEG2-NHS [15 mg (0.03 mmol)], and DIEA [16 ul (0.09 mmol)] and then purged with Argon. After stirring overnight at room temperature, the mixture was rotary evaporated to dryness. The residue was purified by reverse phase prep chromatography, using a gradient of 0.1% TFA water and Acetonitrile, affording 12 (26 mg) in 80% yield. Purity of all compounds were confirmed via LCMS.

## Method and Analysis Details

### *In vitro* studies

#### Stress granule aggregation in SH-SY5Y cells overexpressing TDP-43 SH-SY5Y cells:

A TDP-43 cellular model was successfully generated by establishing stable cell-lines over-expressing wild-type TDP-43 and TDP-43 M337V mutation that causes ALS. Following arsenite treatment, the latter cells display stress granules and TDP-43 aggregates. SH-SY5Y tet-on TDP-43 partial 3’-UTR (wt or M337V), 50.000 cells (DMEM/F12 + 1 μg/mL doxycycline) incubated on cover slips overnight. Cells were treated sodium arsenite at a final concentration of 250 μM and incubated for 90 min. Cells were washed with 500 μL PBS. Cells were fixed with 4% PFA in PBS pH 7.4 for 15 min at RT. Cells were washed with 500 μL PBS. Permeabilize/ block cells with 5% milk powder, 1% BSA and 0.5% saponin in PBS for 45min @ RT. Add respective {TDP-43 C-terminal domain Antibody (1:1000; Purchased from Proteintech), HuR (1:500; Purchased from Santa Cruz} in 1% PBS and 0.5% saponine in PBS and incubated overnight at 4°C. Cells were washed 3x with 500 μL PBS and incubated with secondary antibody at a dilution of 1:1000 AlexaFluor a-rabbit 594 (highly cross-adsorbed) (Thermo Fisher) or AlexaFluor a-mouse 488 (highly cross-adsorbed) (Thermo Fisher). Cells were washed 3x with 500 μL PBS, then cells were washed with 500 μL dH_2_O. ProLong Gold with DAPI embedding medium was used to fix cells on a glass slide. Image collection was done on a Zeiss AxioImager 2 equipped with an Apotome using the following filtersets. For the automated image analysis, each raw grayscale channel image was saved and analyzed independently. Each image set was calibrated using the TDP-43 induced, sodium arsenite treated condition as a reference for exposure time of the different channels.

#### High-content imaging of endogenous TDP-43 in stress granules (SGA):

On day 1, seed 20,000 patient derived fibroblasts per well in a 24 well glass bottom plate or 6000 cells per well in a 96 well plate. On day 2, sonicate compounds for 10 mins at 37°C before use. Add compounds at the desired final concentration in fresh media to the respective wells. Add equivalent amount of DMSO (LC-MS grade) to control wells. On Day 3, add sodium arsenite treatment- Add sodium arsenite at a final concentration of 500uM. Incubate at 37°C for 60 mins. Wash 1X with PBS and fix cells with 4% para formaldehyde (in PBS, prepared freshly, methanol free) for 15 mins at room temperature.

Wash 3x with PBS. Permeabilization and Blocking- Add 0.1% Triton-X for 10 mins for permeabilization followed by 1 hour of blocking in 1% BSA. Immunostaining- Add the following primary antibodies in 1% BSA (in PBS) and incubate it overnight at 4°C. Rabbit polyclonal TDP-43 C-terminal antibody (Proteintech 12892-1-AP)- 1:450; mouse monoclonal HuR antibody (Santa Cruz sc-5261)- 1:500. On day 4, wash 3x with PBST (PBS + 0.1% Tween). The following secondary antibodies from Thermofisher Scientific (1:500) in 1% BSA (in PBS) and keep it in dark for 1-2 hours at room temperature.

Alexa 594 anti-rabbit (highly cross-adsorbed); Alexa 488 anti-mouse (highly cross-adsorbed); Wash 3x with PBST in dark. Add DAPI in PBS for nuclear staining. Imaging and Image analysis is done as explained below. In brief, the immuno-stained cells were imaged with Nikon Ti inverted fluorescence microscope having CSU-22 spinning disk confocal and EMCCD camera. Plan Apo objectives and NIS-Elements AR software were used for image acquisition. At least 30-50 images per well is taken.

#### Nucleocytoplasmic assay (NCA) in FTD PDFs:

Skin-derived fibroblasts cells from a sporadic Frontotemporal Degeneration (FTD) and from individuals with VCP mutation R191Q (familial FTD) and TDP-43 mutation G298S (familial ALS) acquired from the National Institute of Neurological Disorders and Stroke (https://stemcells.nindsgenetics.org/), were grown in HyClone DMEM High Glucose (GE Healthcare Life Sciences) supplemented with 15% FBS and 1% NEAA (Non-Essential Amino Acids), at 37°C in an humidified atmosphere of 5% CO_2_. On Day 1, seed 600 cells per well in a 96 well glass bottom plate or 1200 cells per well in a 24 well glass bottom plate. Incubate for 4 days, at 37°C in a humidified atmosphere of 5% CO_2_. On day 5, sonicate compounds for 10 mins at 37°C before use. Add compounds at the desired final concentration in fresh media to the respective wells. Add equivalent amount of DMSO (LC-MS grade) to control wells. Incubate for 4 days, at 37°C in a humidified atmosphere of 5% CO_2_. On day 9, wash 2x with PBS. To fix add 4% para formaldehyde (in PBS, prepared freshly, methanol free) for 15 mins at room temperature. Wash 3x with PBS. Blocking and permeabilization- Add 1% BSA + 1% saponin (prepared in PBS) for 1 hour.

Immunostaining- Add the following primary antibodies in 1% BSA (in PBS) and incubate it overnight at 4°C. Rabbit polyclonal TDP-43 C-terminal antibody (Proteintech 12892-1-AP)- 1:350; mouse monoclonal HuR antibody (Santa Cruz sc-5261)- 1:500. On day 10, wash 3x with PBST (PBS + 0.1% Tween). Add the following secondary antibodies from Thermofisher Scientific (1:500) in 1% BSA (in PBS) and keep it in dark for 1-2 hours at room temperature. Alexa 594 anti-rabbit (highly cross-adsorbed). Alexa 488 anti-mouse (highly cross-adsorbed). Wash 3x with PBST in dark. Add DAPI in PBS for nuclear staining.The immuno-stained cells are imaged with Nikon Ti inverted fluorescence microscope having CSU-22 spinning disk confocal and EMCCD camera. Plan Apo 20x/0.75 objective and NIS-Elements AR software were used for image acquisition. At least 15 images per well are taken. The exposure times for TDP-43 and HuR must remain constant across one experiment. Each image acquired (in .nd format) is exported into three individual channel images (for DAPI, TDP-43, HuR) in .tiff format. The images are analyzed by the open source image analysis software Cell Profiller. The DAPI image is used to count the total number of cells. The TDP-43 and HuR images are used to count the number of cells containing TDP-43 and/or HuR nuclear staining.

#### Automated image analysis and machine learning tools:

The immuno-stained cells were imaged with Nikon Ti inverted fluorescence microscope having CSU-22 spinning disk confocal and EMCCD camera. Plan Apo objectives and NIS-Elements AR software were used for image acquisition. At least 30-50 images per well is taken. The exposure times for TDP-43 and HuR must remain constant across one experiment. Each image acquired (in .nd format) is exported into three individual channel images (for DAPI, TDP-43, HuR) in .tiff format. The images were analyzed by the open source image analysis software CellProfiler 2.1.1 (offered by Broad Institute of Harvard and MIT- www.cellprofiler.org). This software contains various modules which can be used to analyze images in different ways. A Cell Profiler pipeline from a few of these modules was established to analyze our images in order to quantify the number of cytoplasmic TDP-43 positive HuR stress granules. The outline of the Cell Profiler pipeline: The three channel images are loaded and named DAPI, TDP-43 or HuR. Identify primary objects- The DAPI image is used to identify the nucleus as an object. Identify secondary objects- The nucleus is used to identify the cell boundary in the TDP-43 image by signal propagation. Identify tertiary objects- Based on the nucleus and the cell boundary, cytoplasm is identified as an object. Mask images- Using the cytoplasm object, the TDP-43 and HuR images are masked such that only cytoplasmic signal will remain. Enhance features: Enhance the signal from TDP-43 and HuR aggregates in cytoplasm for efficient identification of the aggregates. Identify primary objects- The TDP-43 aggregates in the cytoplasm were identified from the TDP-43 image and HuR aggregates were identified from the HuR image. Relate objects- This module enables the calculation of the number of TDP-43 aggregates which has HuR and vice versa. Export to spreadsheet- This module exports all data into Excel sheets. The final data has to be curated from the Excel sheets generated by Cell Profiler. The outlines for the different objects (nucleus, cytoplasm, aggregates) must be saved to cross-check the proper identification of objects once the analysis is done. At the beginning of analysis of an experiment, few images (from DMSO wells) must be used as training set for Cell Profiler. Based on the training set, the pipeline must be optimized with respect to intensity threshold, algorithm and size parameters for correct identification of primary and secondary objects (nucleus, cytoplasm, aggregates). It is extremely important to optimize this pipeline for every experiment. The pipeline must remain constant with respect to aggregate identification for the analysis of all the images from the same experiment. Finally, we calculate the number of TDP-43 positive HuR stress granules from the Excel sheets generated at the end of Cell Profiler.

#### HIV infectious virus assay:

MT-2 cells were preseeded in 96-well plates in 100 ul of complete RPMI. Multiple concentrations of PAV-951 were serially diluted in DMSO then into an infection media prepared by diluting NL4-3 Rluc virus stock to 400IU/100ul with complete RPMI, which was transferred onto the MT-2 cells with a final MOI of 0.02 and final DMSO concentration of 1% in infected places. One well received DMSO only, instead of PAV-951, and one well received medium only for normalization and background collection. Cells were incubated at 37°C for 96 hours. 100ul of medium was removed and discarded and 10ul of 15 uM EnduRen luciferase substrate was added to each well, followed by incubation for 1.5 hours at 37°C. Plates were read on a luminescence plate reader. Bioluminescence intensity was read on a Synergy H1 BioTek plate reader. Averages and standard deviation for viral titer observed under different treatment conditions were calculated in Microsoft Excel and graphed as the percent inhibition in PAV-951 treated cells compared to untreated cells.

#### Drug resin affinity chromatography:

Mouse brains from wildtype or SOD1 mutant animals were homogenized in cold phosphate buffered saline (PBS) (10 mM sodium phosphate, 150 mM sodium chloride pH 7.4), then spun at 1,000 rpm for 10 minutes until pelleted. The PBS was decanted and the pellet resuspended in a low salt buffer (10 mM HEPES pH 7.6, 10 mM NaCl, 1 mM MgAc with 0.35% Tritonx100) then centrifuged at 10,000 rpm for 10 minutes at 4°C. The post-mitochondrial supernatant was removed and adjusted to a concentration of approximately 10 mg/ml and equilibrated in a physiologic column buffer (50 mM Hepes ph 7.6, 100 mM KAc, 6 mM MgAc, 1 mM EDTA, 4 mM TGA). In some conditions, the extract was supplemented with an energy cocktail (to a final concentration of 1 mM rATP, 1 mM rGTP, 1 mM rCTP, 1 mM rUTP, and 5 ug/mL creatine kinase). 30 ul or 230 ul of extract was then incubated for one hour at either 4°C or 22°C degrees on 30 ul of affigel resin coupled to THIQ compound or a 4% agarose matrix (control). The input material was collected and the resin was then washed with 3 ml column buffer. The resins were eluted for 2 hours then overnight at 22°C then 4°C in 100 ul column buffer containing 100 uM of the cognate compound. Eluates were run on western blot or sent for mass spectrometry for analysis.

#### Chemical photocrosslinking:

Extract from mouse brain and PDFs grown in minimum essential media were prepared as above then adjusted to a protein concentration of approximately 3 mg/ml in column buffer containing 0.01% triton. 1% DMSO or 100 uM PAV-073 was added to 6 ul of extract, then 3 uM of PAV-073 photocrosslinker or a negative control crosslinker (comprising of the biotin and diazirine moieties without compound) were added. The extract was incubated for 20 minutes then exposed to UV at 365 nM wavelength for 10 minutes then left on ice for one hour. After crosslinking, samples were divided in two 20 ul aliquots and one set was denatured by adding 20 uL of column buffer 4 ul of 10% SDS, 0.5 ul 1 M DTT, and boiling for 5 minutes. Both native and denatured aliquots were then diluted in 800 ul column buffer containing 0.1% triton. 5 ul of magnetic streptavidin beads (Pierce) were added to all samples and mixed for one hour at room temperature to capture all biotinylated proteins and co-associated proteins. Samples were placed on a magnetic rack to hold the beads in place and washed three times with 800 ul of column buffer containing 0.1% triton. After washing, beads were resuspended in 80 ul of gel loading buffer containing SDS and analyzed by western blot or blot for affinity purified streptavidin. Samples were analyzed by western blot.

#### Western blotting:

SDS/PAGE gels were transferred in Towbin buffer (25 mM Tris, 192 mM glycine, 20% w/v methanol) to polyvinylidene fluoride membrane, blocked in 1% bovine serum albumin (BSA) in PBS, incubated overnight at 4°C in a 1:1,000 dilution of 100 ug/mL affinity-purified primary IGG to PDI in 1% BSA in PBS containing 0.1% Tween-20 (PBST). Membranes were then washed twice in PBST and incubated for two hours at room temperature in a 1:5000 dilution of secondary anti-rabbit or anti-mouse antibody coupled to alkaline phosphatase in PBST. Membranes were washed two more times in PBST then incubated in a developer solution prepared from 100 uL of 7.5 mg/mL 5-bromo-4-chloro-3-indolyl phosphate dissolved in 60% dimethyl formamide (DMF) in water and 100 ul of 15 mg/ml nitro blue tetrazolium dissolved in 70% DMF in water, adjusted to 50 mL with 0.1 Tris (pH 9.5) and 0.1 mM magnesium chloride. Membranes were scanned and the integrated density of protein band was measured on ImageJ. Averages and the standard deviation between repeated experiments were calculated and plotted on Microsoft Excel.

#### Tandem mass spectrometry (MS-MS):

Samples were processed by SDS PAGE using a 10% Bis-tris NuPAGE gel with the 2-(N-morpholino)ethanesulfonic acid buffer system. The mobility region was excised and washed with 25 mM ammonium bicarbonate followed by 15 mM acetonitrile. Samples were reduced with 10 mM dithoithreitol and 60°C followed by alkylation with 50 mM iodoacetamide at room temperature. Samples were then digested with trypsin (Promega) overnight (18 hours) at 37°C then quenched with formic acid and desalted using an Empore SD plate. Half of each digested sample was analyzed by LC-MS/MS with a Waters NanoAcquity HPLC system interfaced to a ThermoFisher Q Exactive. Peptides were loaded on a trapping column and eluted over a 75 uM analytical column at 350 nL/min packed with Luna C18 resin (Phenomenex). The mass spectrometer was operated in a data dependent mode, with the Oribtrap operating at 60,000 FWHM and 15,000 FWHM for MS and MS/MS respectively. The fifteen most abundant ions were selected for MS/MS.

Data was searched using a local copy of Mascot (Matrix Science) with the following parameters: Enzyme: Trypsin/P; Database: SwissProt Human (conducted forward and reverse plus common contaminants); Fixed modification: Carbamidomethyl (C) Variable modifications: Oxidation (M), Acetyl (N-term), Pyro-Glu (N-term Q), Deamidation (N/Q) Mass values: Monoisotopic; Peptide Mass Tolerance: 10 ppm; Fragment Mass Tolerance: 0.02 Da; Max Missed Cleavages: 2. The data was analyzed by spectral count methods.

### *In vivo* studies

#### Transgenic human TDP-43 mutant *C. elegans*:

MosSCI homologous-recombination transgenesis was used to create an unc- 47p::hTDP-43::unc-54 utr or unc-47p::hTDP-43(mutant M337V)::unc-54 utr transgenic. Transgenesis requires MOSSCI plasmid inserted with unc-47p::hTDP- 43::unc-54 utr or unc-47p::hTDP-43(mutant M337V)::unc-54 utr. Injection mix used Standard MosSCI mix. Injections were performed into mos1 ttTi5605 background strain. Extrachromosomal array lines were isolated. Crawling transgenics screened as non-red homozgotes were verified by PCR for insertion/replacement at target locus resulting verified single copy integrated strains. Transgenic *C. elegans* expressing the human TDP-43 wild-type or mutant TDP-43 M337V animal model that mimic aspects of TDP-43 specific ALS disease pathogenesis were generated. The transgenic *C. elegans* had a single copy of the human TDP-43 gene integrated into its genome. The expression is controlled by an unc-47 promoter and hence human TDP-43 protein was specifically expressed only in the *C. elegans* motor neurons. *C elegans* studies were also performed with worms transgenic for the hTDP-43 (A315T) mutation using methods described in detail elsewhere [24,58].

#### Age-synchronizing *C. elegans*:

Filtered deionized water was used to wash worms off of plates and into 15 ml tubes which were centrifuged at 1200 rpm for 2 minutes three times. The supernatant was aspirated and 5 ml of NaOH+ bleach solution added. This was vortexed gently about every minute and monitored by microscope. The adults worms split open and their eggs were released. The adult worms also dissolved into the solution. Once all adult worms dissolved, the reaction was neutralized by adding 5 ml of M9 buffer followed by three rounds of centrifugation at 2500 rpm for 2 minutes. After one wash with 10 ml of water, all but about 200-1000 ul was aspirated from the 15 ml tube and the remaining pellet was re-suspended in leftover water. This was dropped onto the plates evenly, thus ensuring that the larva that hatches had enough food while they grew over the next few days. Plates were stored at 20°C.

#### Swimming-induced paralysis (swip) assay:

The age-synchronized worms were washed off NGM plates in S-media that contains 0.02% Triton. This allows for a more consistent number of worms while pipetting, as less worms stick to the plastic pipette tips. The volume is adjusted with S-media until there would be 60-70 worms per 20 ul. Worms were scored as paralyzed if their body cannot make a bending “S” movement. Paralyzed worms can often still make small movements with their head or tail. Videos are captured using a Lumenera Infinity 3s camera fitted to a Nikon TE300 microscope at 2x magnification and recorded to ImageJ. In some experiments videos are captured using Phylumtech’s Wormtracker machine. The videos were analyzed using ImageJ *C. elegans* motility analysis software. Level of activity were denoted based on improvement in swimming induced paralysis (SWIP) in human TDP-43 transgenic *C. elegans* disease model. The automation data measuring paralysis measured average body bends per second of a population. Improvement in SWIP from control in the population of worms was observed.

#### *Drosophila* drug feeding assay:

Melt cornmeal-molasses-yeast fly food was mixed with certain concentrations of compound at high temperature and cooled to RT. 0.1% DMSO was used as the vehicle control. Parent flies were crossed on food supplemented with drugs and the offspring were raised on the same food. Adult flies were aged on the drug-containing food for 15 days before analyzing their eye morphology. For quantification of outer eye morphological defects, ten flies were quantified.

#### SODG93A mouse efficacy study:

Wildtype and SODG93A mutant mice (B6.CgTg(SOD1*G93A)Gur/J) were bred and maintained at the Jackson Laboratories breeding facility, grown for 5 weeks, then given daily IP doses with vehicle, compound T18, or compound T20 for another 5 weeks. Weight and serum pNHF were tracked during the study. pNFH serum levels were measured through the ELLA microfluidic ELISA platform (Protein Simple) according to the manufacturer’s instructions. Briefly, a maximum of 100 μl blood was collected through facial vein puncture at the indicated time points. Serum samples were prepared by centrifugation of BD Vacutainer SST tubes (BD Diagnostics) and stored at −80°C until used.

## Supplementary Material

JEN-24-103-Supplimentary-file

## Abbreviations

ALS: Amyotrophic Lateral Sclerosis
rATP: Adenosine Triphosphate
ANOVA: Analysis of Variance
BSA: Bovine Serum Albumin
CFPSA: Cell-Free Protein Synthesis and Assembly
Cl: Chloride
c9orf72: Chromosome 9 open reading frame 72 (c9orf72)
rCTP: Cytidine Triphosphate
Cyt: Cytoplasm
DAPI: 4’,6-diamidino-2-phenylindole
DAVID: Database for Annotation, Visualization, and Integrated Discovery
DMSO: Dimethylsulfoxide
EMCCDs: Electron-multiplying CCDs
eDRAC: Energy-dependent Drug Resin Affinity Chromatography
FTD: Fronto-Temporal Dementia
GO: Gene Ontology
rGTP: Guanosine Triphosphate
EC50: Half Maximal Effective Concentration
HPLC: High Pressure Liquid Chromatography
HUR: Human Antigen R
HTLV-1: Human T-cell Leukemia Virus Type 1
MT-2: Infected Cell Line
HIV: Human Immunodeficiency Virus
IP: Intraperitoneal
KAP1: KRAB-associated Protein 1
LC: Liquid Chromatography
Mg: Magnesium
MS: Mass Spectrometry
MIF: Macrophage Migration Inhibitory
mL: Milliliter
mM: Millimolar
mg: Milligram
Nuc: Nucleus
PDF: Patient-derived Fibroblasts
K: Potassium
PBS: Phosphate Buffered Saline
pNFH: Phosphorylated Neurofilament Heavy Chain
PPDI: Protein Disulfide Isomerase
Pt: Patient
SEM: Standard Error of Measurement
p62/SQSTM1: Sequestosome 1
SOD: Superoxide Dismutase
MTE: SOD1 G93A Mutant THIQ Eluate
SAP: Streptavidin Precipitation
SG: Stress Granule
SAR: Structure-activity Relationship
SWIP: Swimming-induced Paralysis
MS-MS: Tandem Mass Spectrometry
THIQ: Tetrahydroisoquinoline
TDP-43: Transactive DNA-binding Protein of 43 kDa
TRIM-28: Tripartite Motif-containing Protein 28
UV: Ultraviolet
UTP: Uridine Triphosphate
VCP: Valosin-containing Protein
WT: Wild-type
WTE: WT THIQ Eluate
