# Supplementary material for: Protein Assembly Modulation: A New Approach to Amyotrophic Lateral Sclerosis (ALS) Therapeutics
Source: J Exp Neurol. Author manuscript; Available in PMC 2025 Sep 19. (PMC12445735; doi:10.33696/Neurol.5.103)
Supplement: JEN-24-103-Supplimentary-file [file NIHMS2109961-supplement-JEN-24-103-Supplimentary-file.zip › JEN-24-103_Supplementary_File/JEN-24-103_Supplementary_Figure_Legends.docx]

**Supplemental Figure Legends**

**Supplemental Figure 1. Synthesis and activity of PAV-073. Supplemental Figure 1A** shows the synthetic scheme for PAV-073. **Supplemental Figure 1B** shows activity of PAV-073 against infectious HIV. MT-2 cells were infected with NL4-3 Rluc HIV and treated with PAV-073 for four days. Averages and standard deviation of viral titer observed with triplicate repeated dose-titrations of PAV-073 are shown as a percentage of the titer observed in DMSO-treated cells. Statistical significance was calculated on GraphPad Prism using ordinary one-way ANOVA and Dunnett’s multiple comparisons test. **Supplemental Figure 1C** shows activity of PAV-073 relative to pimozide (40 uM) in ameliorating the condition of transgenic *C. elegans* expressing the human TDP-43 A315T mutation. Nematodes were age-matched and grown on standard nematode grown media plates until day 1 of adulthood at which point they were collected and placed in 96 well plates (50-70 animals per well) and treated with compound or control. Animal movement was then tracked for 30 minutes using WMicroTracker ONE.

**Supplemental Figure 2.** **Stress granule induction and quantitation. Supplemental Figure 2A** shows immunostain for DAPI, HuR, and TDP-43 in PDFs treated with 500 uM sodium arsenite for one hour. **Figure 2B** shows how cell profiler imaging was used to identify the number of TDP-43 positive HuR aggregates per cell.

**Supplemental Figure 3. A.** Shown are three diverse ALS patients without a family history of ALS. Fibroblast lines were developed from each of the three patients and assessed by either the NCA or SGA assays described in Figure 1 upon treatment with vehicle or assembly modulator compound T6. **B.** by NCA Pt #13 has massive mislocalization and a robust response, Pt#14 has modest mislocalization with a correspondingly modest response and Pt #11 has no detectable mislocalization. **C.** The same patient-derived fibroblasts were assessed by the SGA assay. Here, Pt#13 and #14 show only a modest SGA response. Pt #11 however shows a robust response. This suggests that the heterogeneity observed between ALS patients resides in part, in the targets of assembly modulators. While different patients have defects primarily observed by one or the other assay, both assays respond to assembly modulator compound T6 treatment.

**Supplemental Figure 4.** Moderation of toxicity with THIQ lead series advancement. Compounds of comparable potency but differences in toxicity were identified from the lead series and assessed in transformed cells, ALS PDFs, and iPSC-derived motor neurons. The lead series progression to diminished toxicity is confirmed in all cell lines using both cell TiterGlo and Alamar Blue toxicity assays.

**Supplemental Figure 5.**  Maximum tolerated dose (MTD, PK, 2 week toxicity of T-18 analog. Compound T-18 was synthesized, purified and formulated in 10% DMSO, 455% propylene glycol, 35% phosphate buffered saline pH 7.2 and mice were dosed IP and PO once daily at escalating doses until appearance of toxicity (20mg/kg IP, >40mg/kg PO in mice). PK and organ exposure was determined in mice and rats after a single dose at 10mg/kg (mice) and 20mg/kg (rats) and 10mg/kg IP (rats). Multi-day toxicity was determined in mice dosed IP 10/mg/kg once daily for 14 days. At the indicated times, animals were euthanized, blood and tissues collected and analyzed as presented.

**Supplemental Figure 6.** Mouse toxicity and tissue exposure studies. Maximum tolerated dose (MTD, PK, 2 week toxicity of T-20 analog. Compound T-20 was synthesized, purified and formulated in 10% DMSO, 55% propylene glycol, 35% phosphate buffered saline pH 7.2 and mice were dosed IP and PO once daily at escalating doses until appearance of toxicity (20 mg/kg IP). PK and organ exposure was determined in mice after a single dose at 10mg/kg IP and 2mg/kg IV. Multi-day toxicity was determined in mice dosed IP 10/mg/kg once daily for 14 days. At the indicated times, animals were euthanized, blood and tissues collected and analyzed as presented.

**Supplemental Figure 7. Synthetic scheme for PAV-073 photocrosslinker analog.** Synthetic scheme for the photocrosslinker analog of PAV-073 used to identify PDI as the direct drug binding protein (see **Figure 5**).

**Supplemental Figure 8.** KEGG ALS disease pathway map with proteins from the THIQ eluate shown in purple. Some proteins on the map represent more than one gene or use different names from our dataset. Translations: EAAT2 = SCL1A2; CxV = ATP5A1, ATP5B, ATP5C1, ATP5H; CaN = PPP3CA, PPP3CB; 19S = PSMD2; VCP = VCP; BiP = HSPA5; NEFL = NEFL; NEFM = NEFM; NEFH = NEFH; CAT = CAT; Kinesin1 = KIF5C; TUBA = TUBA1A, TUBA4A; TUBB = TUBB2A, TUBB3, TUBB4A, TUBB4B.

**Supplemental Figure 9. A.** GO Biological processes represented by proteins in the THIQ eluates. Functional annotation clustering analysis of GO biological processes using DAVID for biological processes enriched in the THIQ eluate proteins. Clusters of similar biological themes are represented by different colors, and the bar graph indicates the number of proteins associated with each biological process. The p-value for each biological process is displayed next to its respective bar.

**B.** GO biological processes in upregulated, downregulated and unchanged THIQ eluate protein groups. . Functional annotation clustering analysis of GO biological processes using DAVID separately for downregulated, upregulated, and unchanged protein groups from SE compared to WTE. Enriched biological processes for upregulated proteins are colored green, downregulated in red, and unchanged in yellow. Clusters of similar biological themes are separated by spaces in the graph. The bar graph indicates the number of proteins associated with each biological process, and the p-value is displayed next to each bar.
