# Supplementary figures and images for: Protein Assembly Modulation: A New Approach to Amyotrophic Lateral Sclerosis (ALS) Therapeutics
Source: J Exp Neurol. Author manuscript; Available in PMC 2025 Sep 19. (PMC12445735; doi:10.33696/Neurol.5.103)

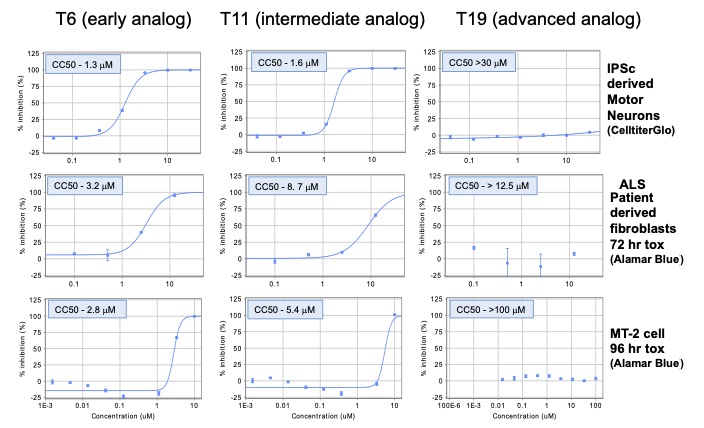

Supplement: JEN-24-103-Supplimentary-file [file NIHMS2109961-supplement-JEN-24-103-Supplimentary-file.zip › JEN-24-103_Supplementary_File/JEN-24-103_Supplementary_Figure 4.jpg]

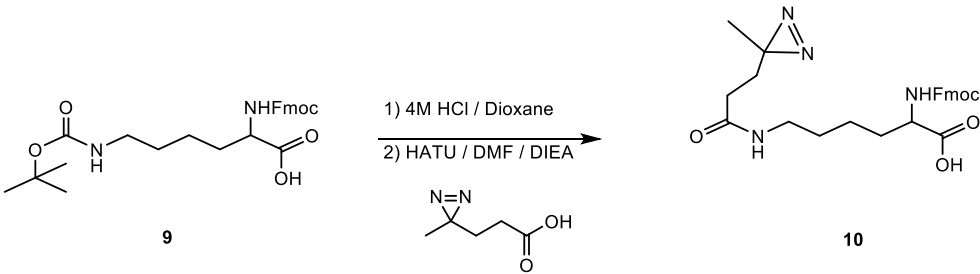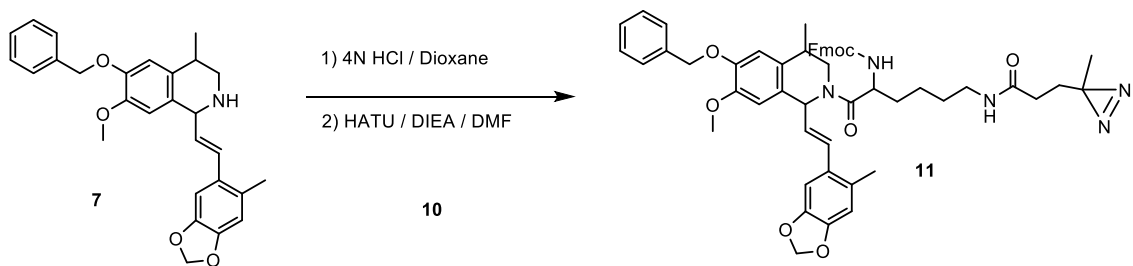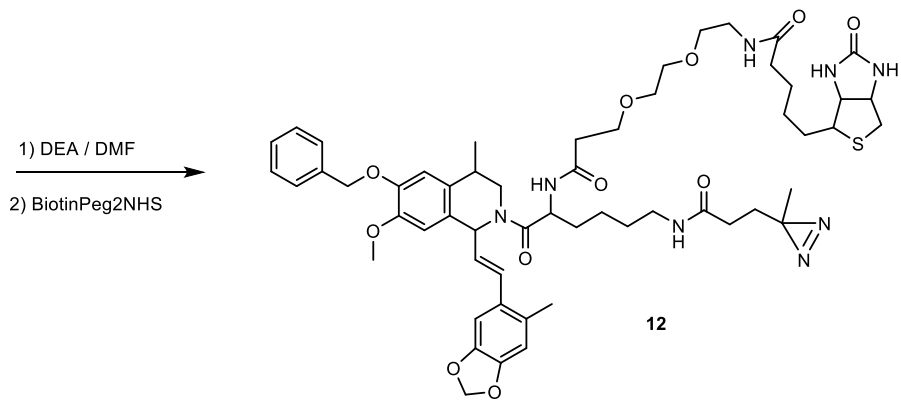

Supplement: JEN-24-103-Supplimentary-file [file NIHMS2109961-supplement-JEN-24-103-Supplimentary-file.zip › JEN-24-103_Supplementary_File/JEN-24-103_Supplementary_Figure 7.pdf]

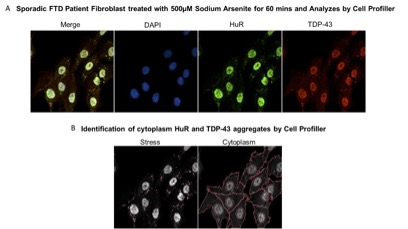

Supplement: JEN-24-103-Supplimentary-file [file NIHMS2109961-supplement-JEN-24-103-Supplimentary-file.zip › JEN-24-103_Supplementary_File/JEN-24-103_Supplementary_Figure 2.jpg]

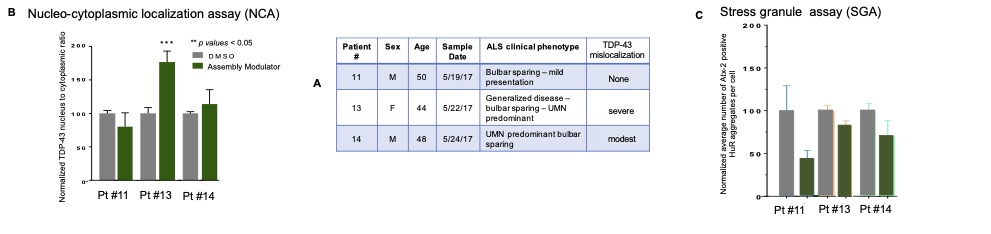

Supplement: JEN-24-103-Supplimentary-file [file NIHMS2109961-supplement-JEN-24-103-Supplimentary-file.zip › JEN-24-103_Supplementary_File/JEN-24-103_Supplementary_Figure 3.jpg]

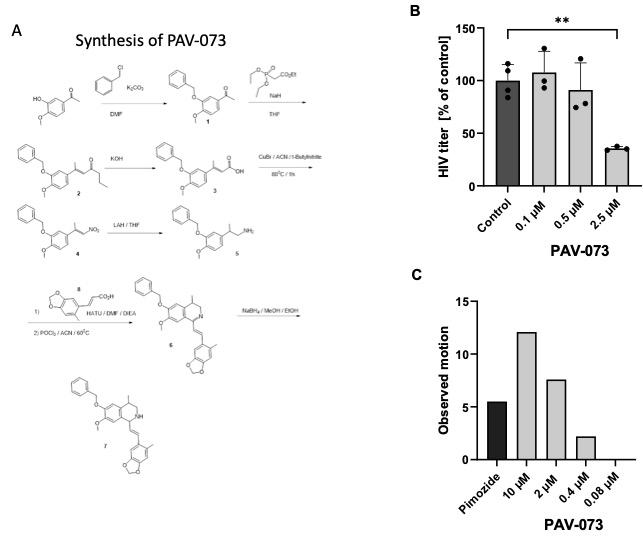

Supplement: JEN-24-103-Supplimentary-file [file NIHMS2109961-supplement-JEN-24-103-Supplimentary-file.zip › JEN-24-103_Supplementary_File/JEN-24-103_Supplementary_Figure 1.jpg]

A

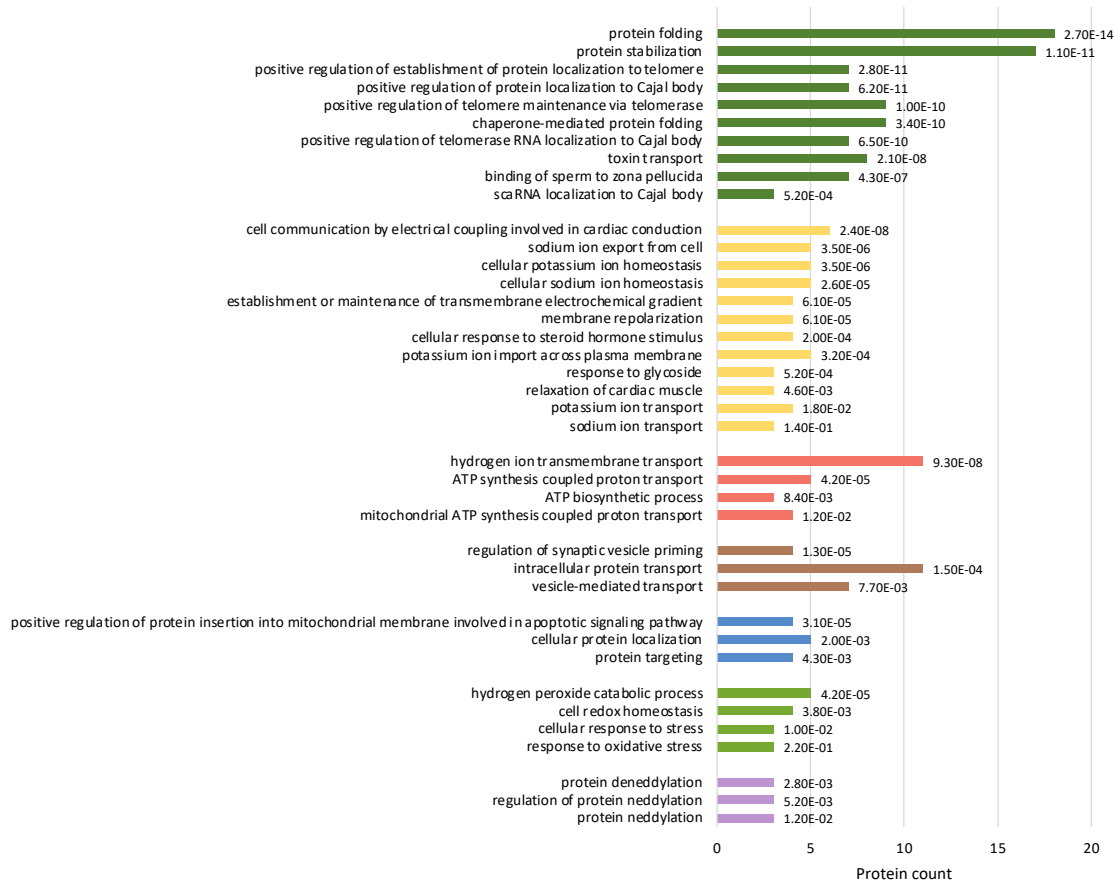

Supplement: JEN-24-103-Supplimentary-file [file NIHMS2109961-supplement-JEN-24-103-Supplimentary-file.zip › JEN-24-103_Supplementary_File/JEN-24-103_Supplementary_Figure 9A.pdf]

B

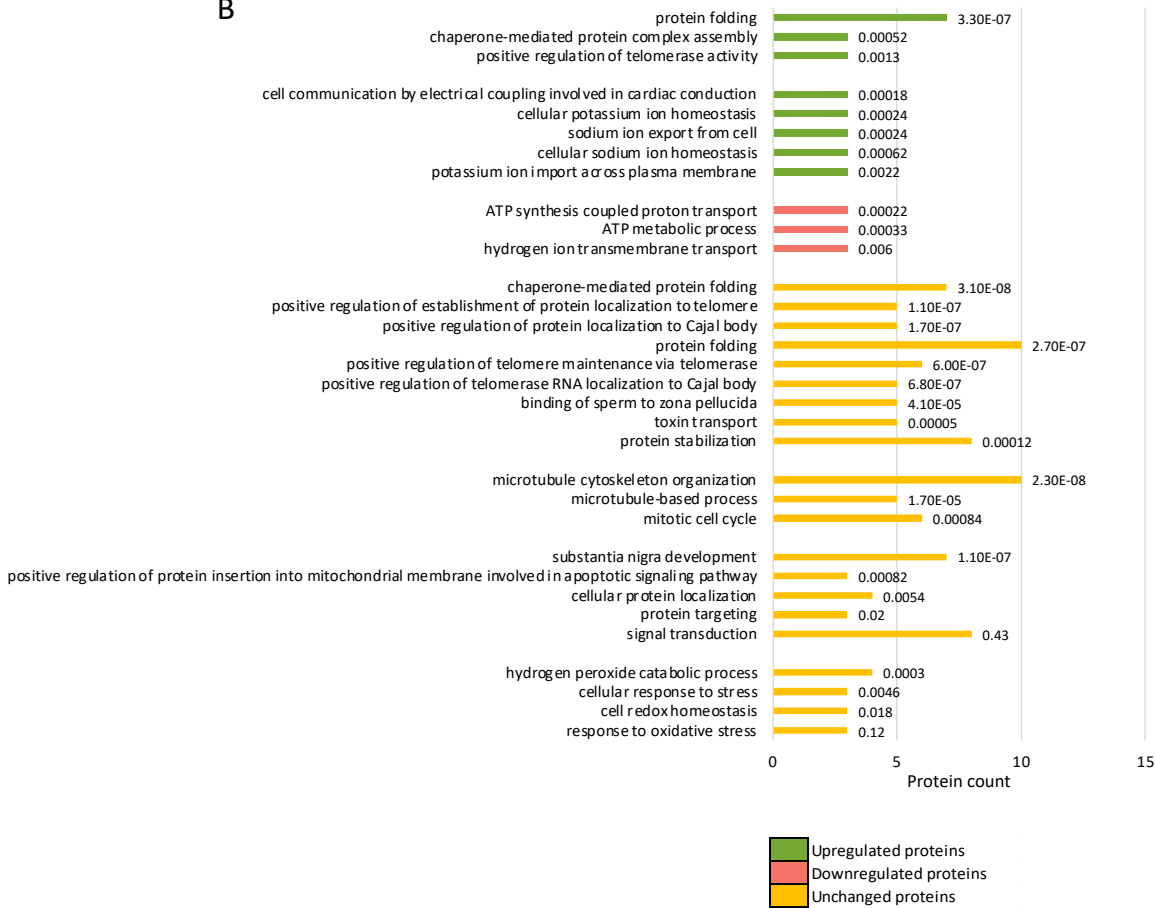

Supplement: JEN-24-103-Supplimentary-file [file NIHMS2109961-supplement-JEN-24-103-Supplimentary-file.zip › JEN-24-103_Supplementary_File/JEN-24-103_Supplementary_Figure 9B.pdf]

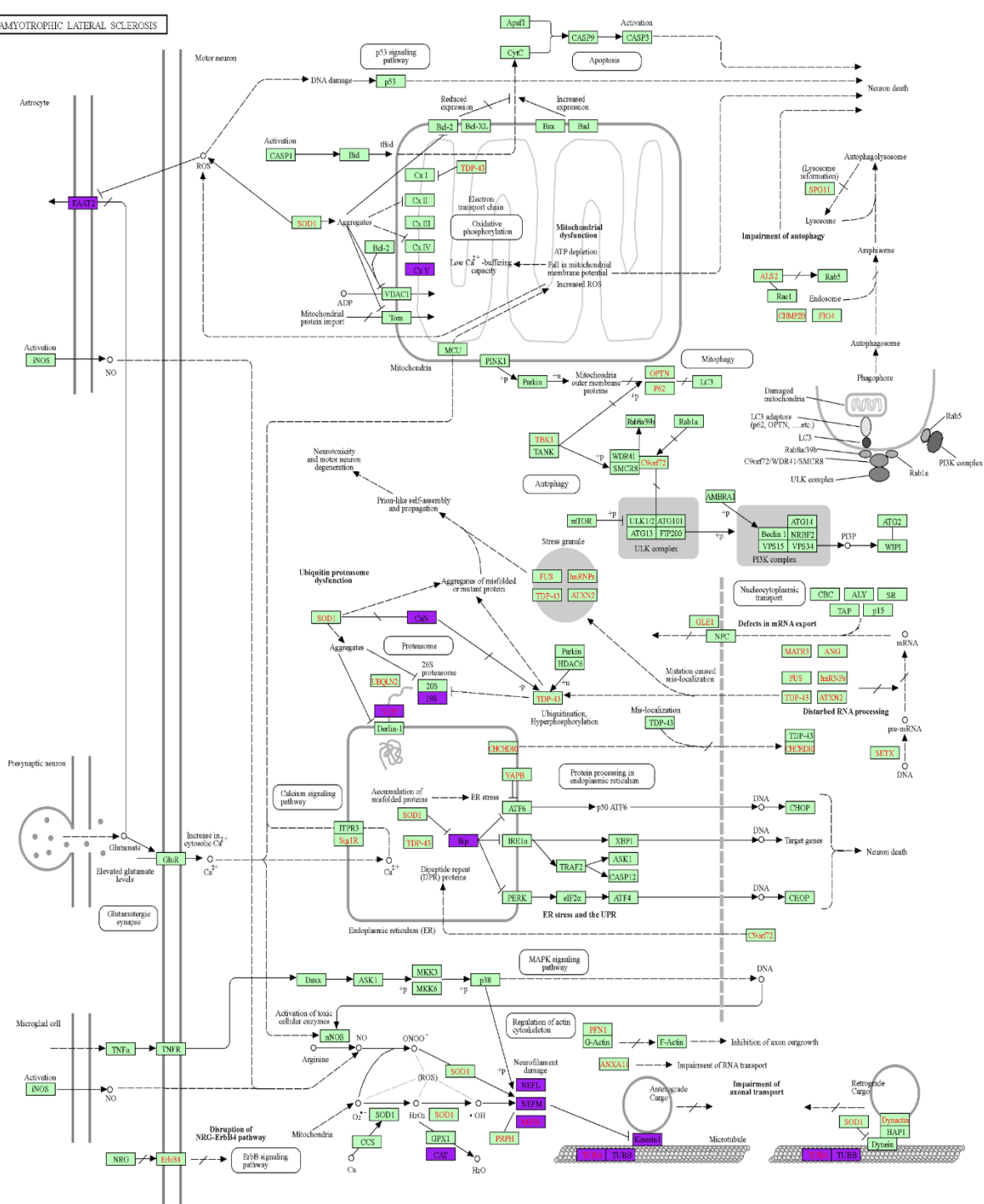

Supplement: JEN-24-103-Supplimentary-file [file NIHMS2109961-supplement-JEN-24-103-Supplimentary-file.zip › JEN-24-103_Supplementary_File/JEN-24-103_Supplementary_Figure 8.pdf]

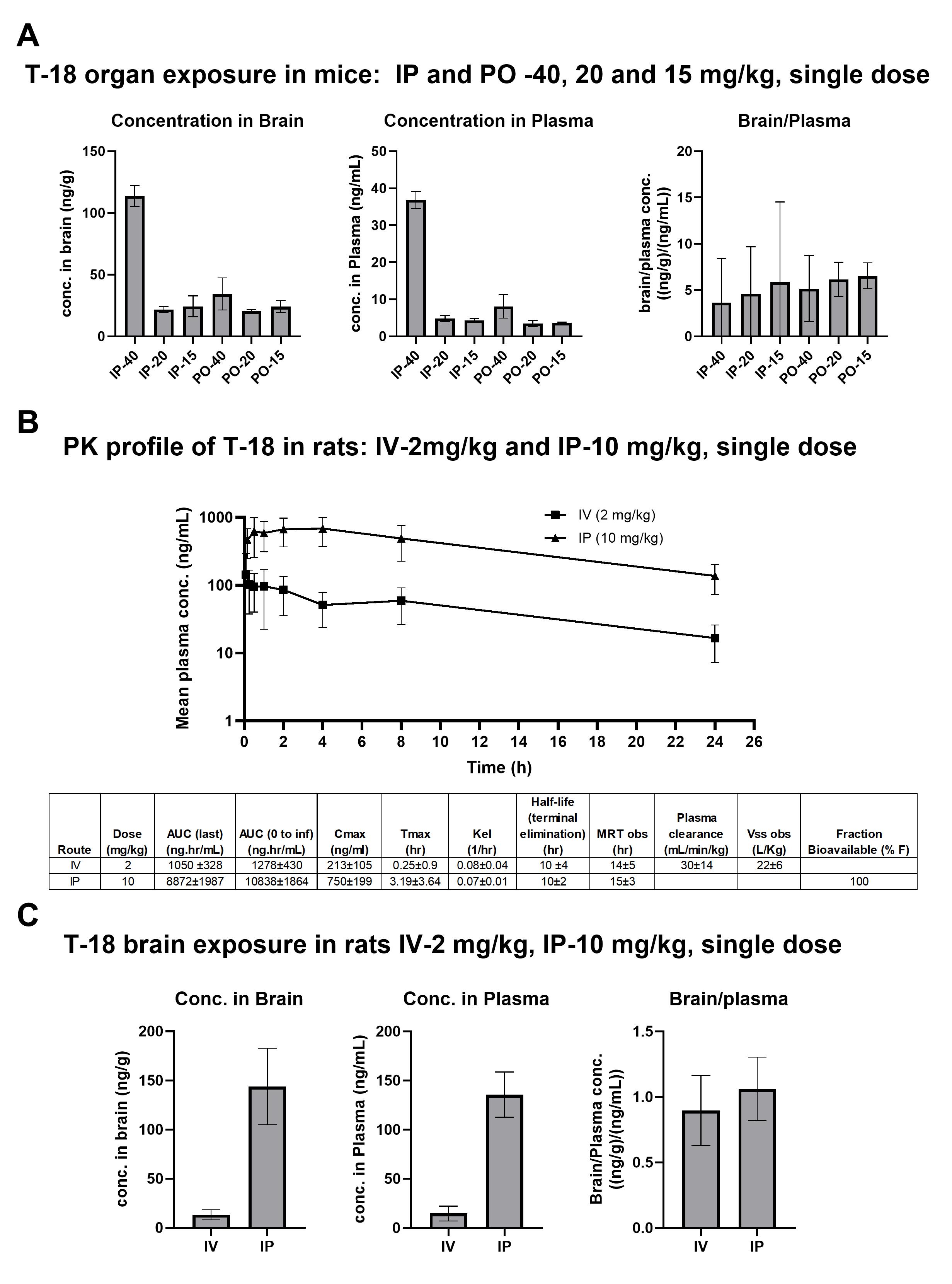

Supplement: JEN-24-103-Supplimentary-file [file NIHMS2109961-supplement-JEN-24-103-Supplimentary-file.zip › JEN-24-103_Supplementary_File/JEN-24-103_Supplementary_Figure 5ABC.jpg]

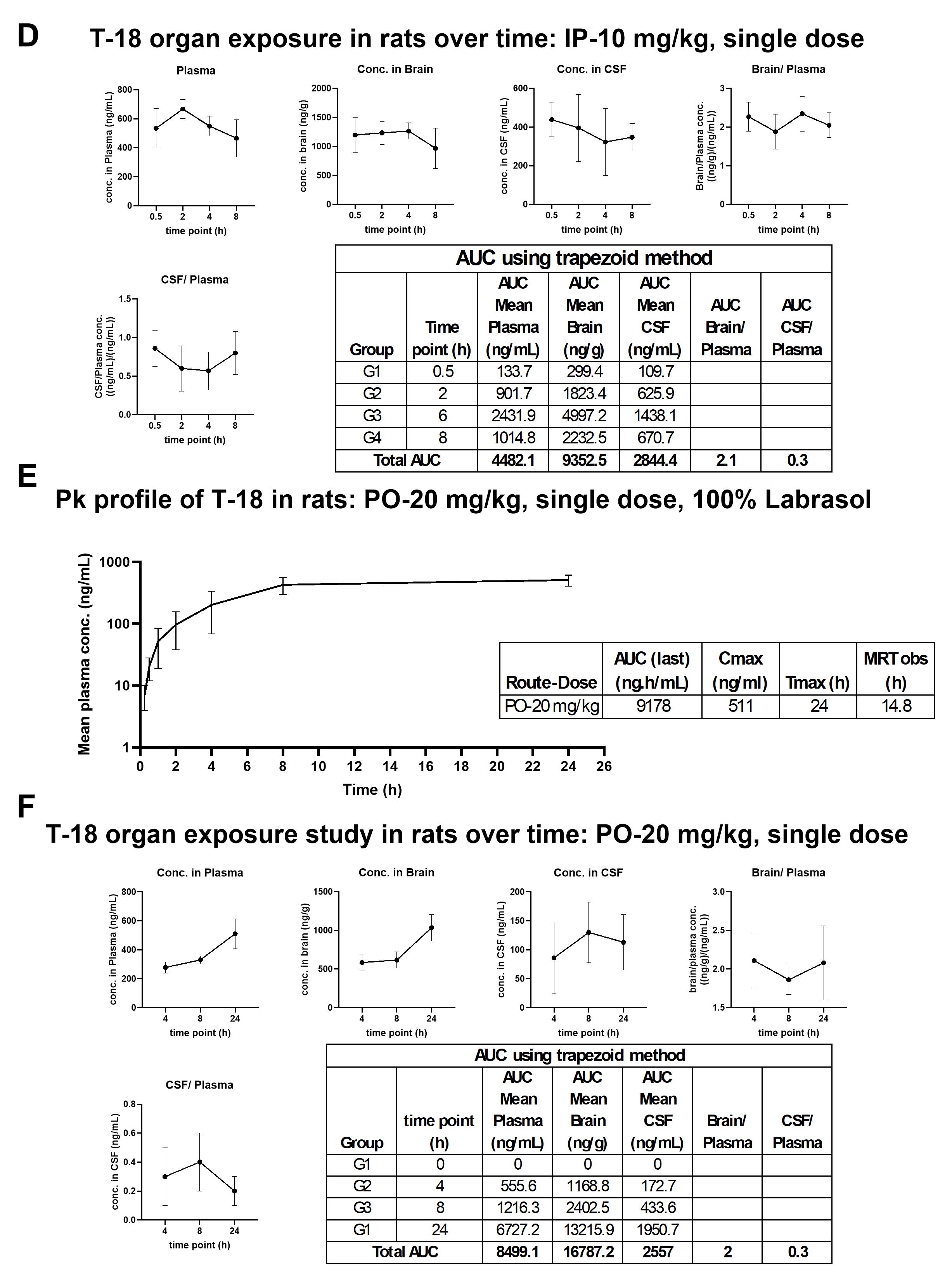

Supplement: JEN-24-103-Supplimentary-file [file NIHMS2109961-supplement-JEN-24-103-Supplimentary-file.zip › JEN-24-103_Supplementary_File/JEN-24-103_Supplementary_Figure 5DEF.jpg]

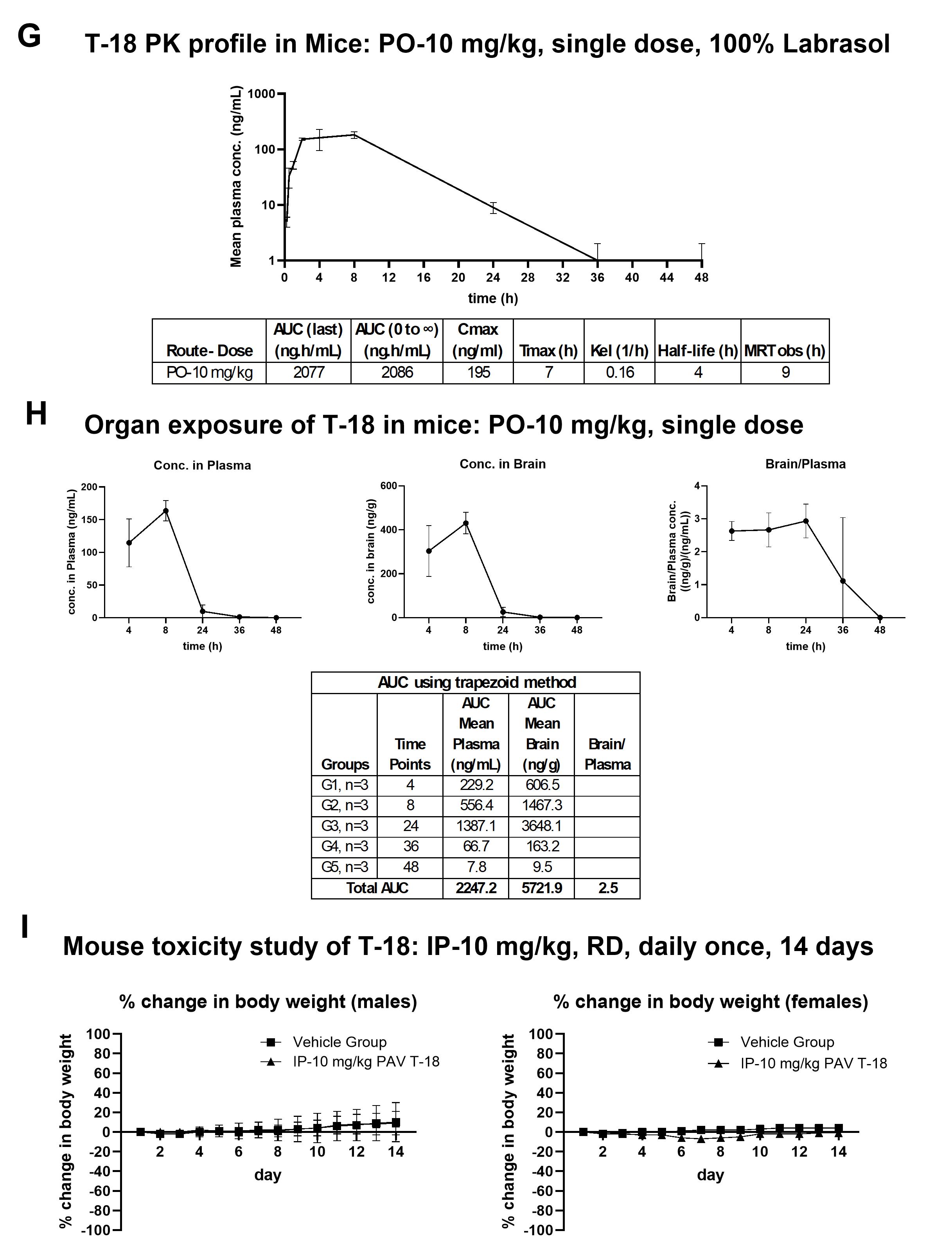

Supplement: JEN-24-103-Supplimentary-file [file NIHMS2109961-supplement-JEN-24-103-Supplimentary-file.zip › JEN-24-103_Supplementary_File/JEN-24-103_Supplementary_Figure 5GHI.jpg]

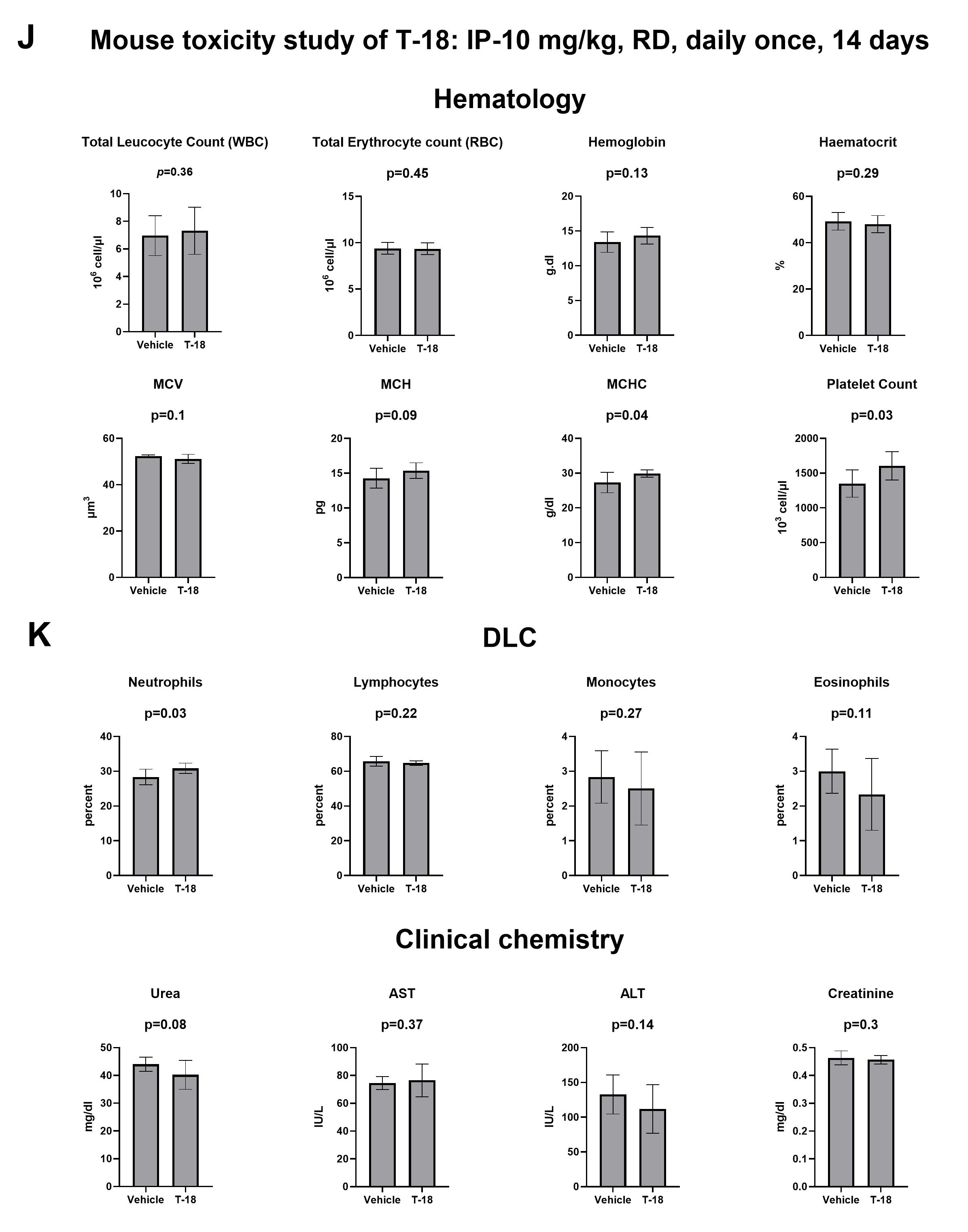

Supplement: JEN-24-103-Supplimentary-file [file NIHMS2109961-supplement-JEN-24-103-Supplimentary-file.zip › JEN-24-103_Supplementary_File/JEN-24-103_Supplementary_Figure 5JK.jpg]

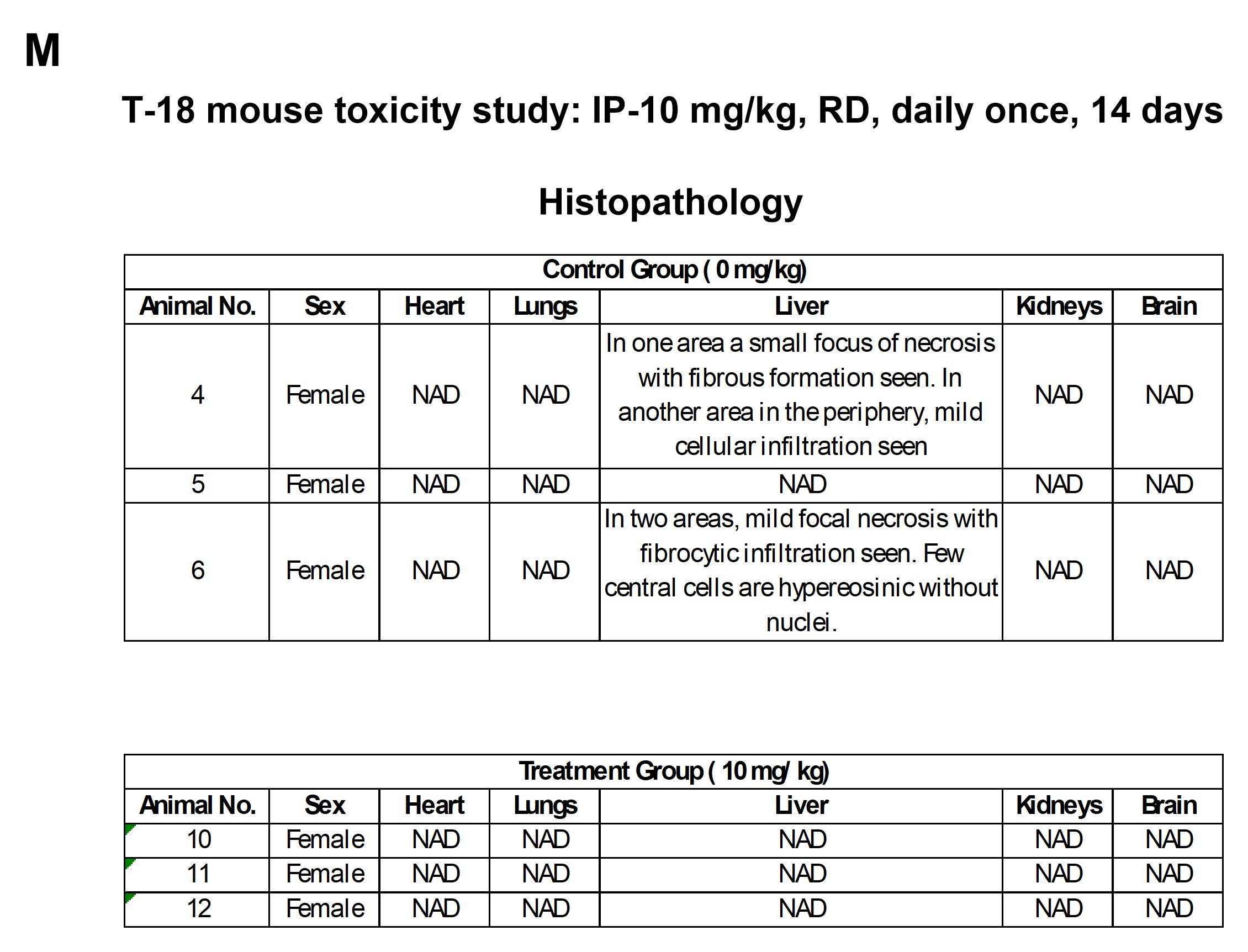

Supplement: JEN-24-103-Supplimentary-file [file NIHMS2109961-supplement-JEN-24-103-Supplimentary-file.zip › JEN-24-103_Supplementary_File/JEN-24-103_Supplementary_Figure 5M.jpg]

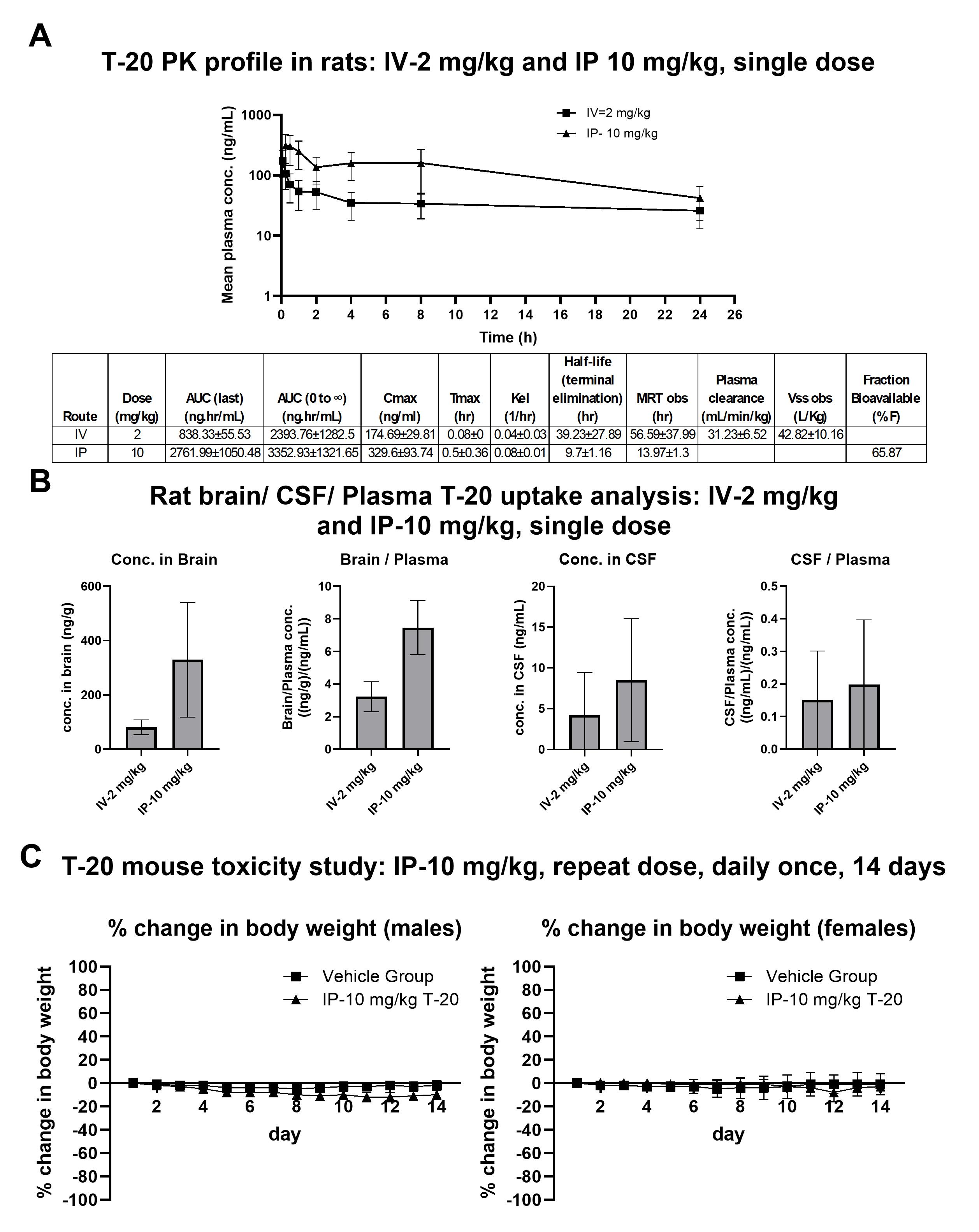

Supplement: JEN-24-103-Supplimentary-file [file NIHMS2109961-supplement-JEN-24-103-Supplimentary-file.zip › JEN-24-103_Supplementary_File/JEN-24-103_Supplementary_Figure 6ABC.jpg]

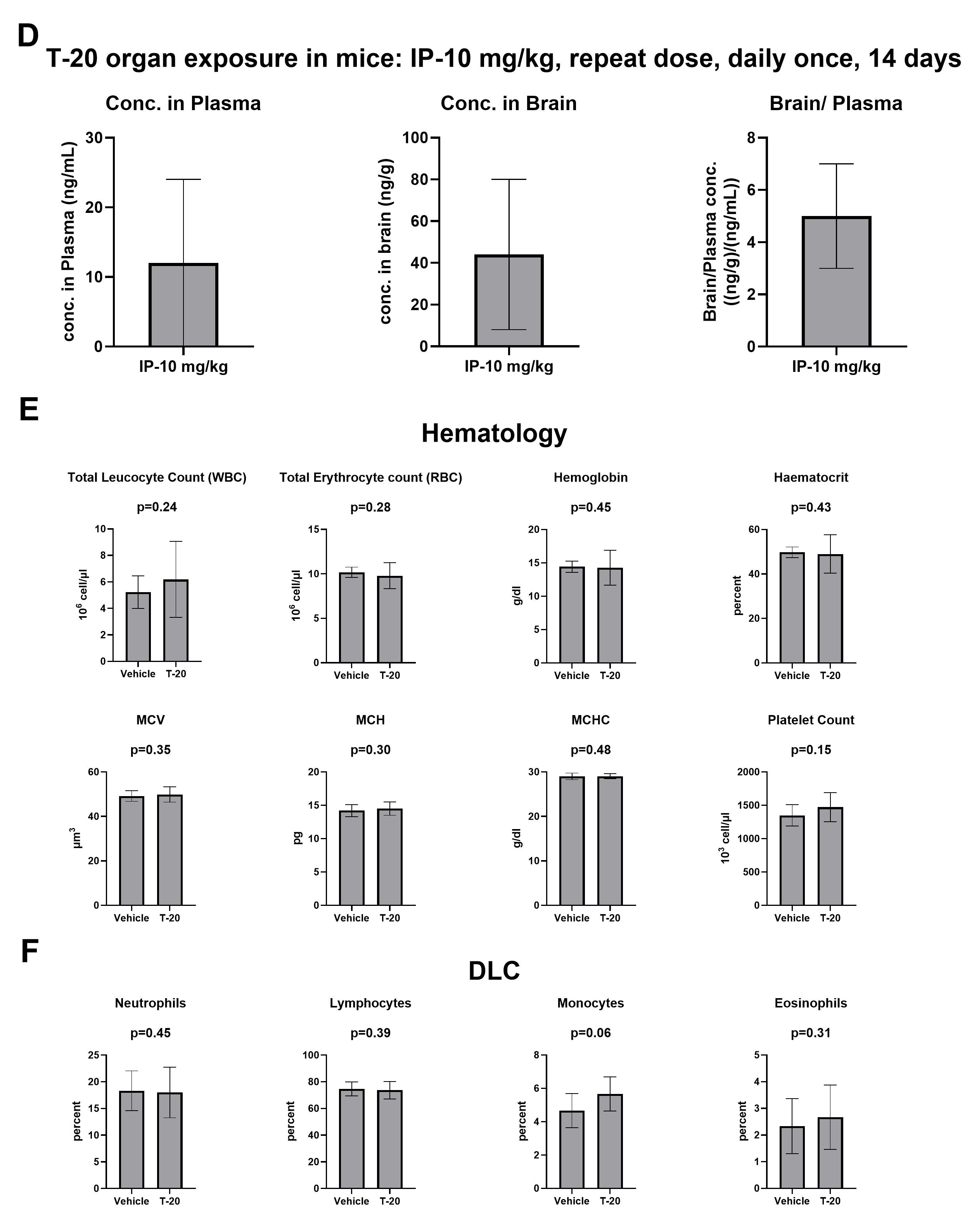

Supplement: JEN-24-103-Supplimentary-file [file NIHMS2109961-supplement-JEN-24-103-Supplimentary-file.zip › JEN-24-103_Supplementary_File/JEN-24-103_Supplementary_Figure 6DEF.jpg]

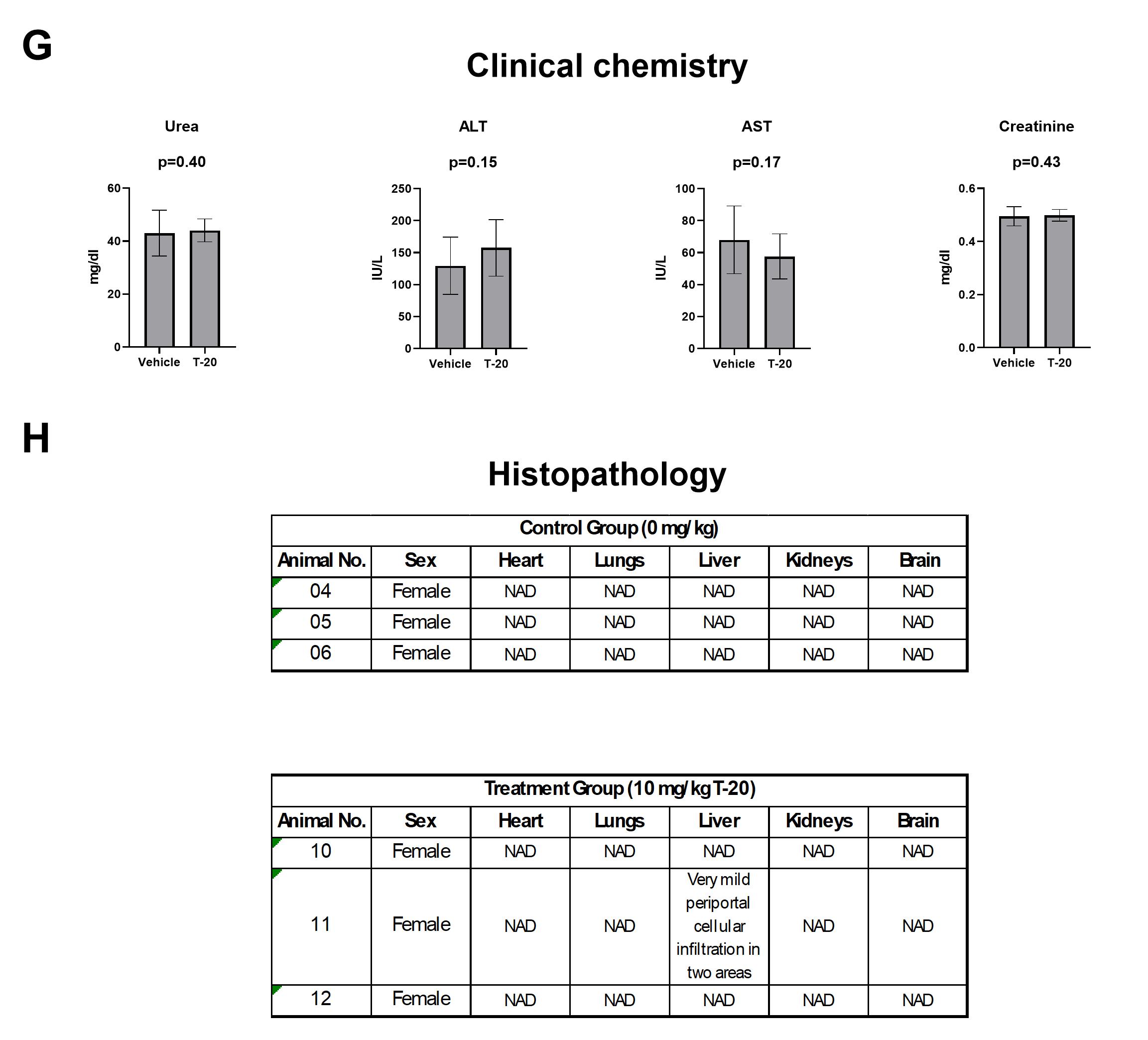

Supplement: JEN-24-103-Supplimentary-file [file NIHMS2109961-supplement-JEN-24-103-Supplimentary-file.zip › JEN-24-103_Supplementary_File/JEN-24-103_Supplementary_Figure 6GH.jpg]
